# Supplementary material for: Donor activity is associated with US legislators’ attention to political issues
Source: PLoS One. 2023 Sep 20;18(9):e0291169. doi: 10.1371/journal.pone.0291169 (PMC10511130; doi:10.1371/journal.pone.0291169)
Supplement: S1 Appendix — (PDF) [file pone.0291169.s001.pdf]

S1 Appendix.

Detailed Data Overview.

A brief overview of our new database is provided in S1 Fig.

A common primary ID used in all the relations included in the US congressional record database (containing floor speeches and legislator information such as their state, party, committee assignments, etc.) is the 'BioGuide ID' (*bioguide_id*) (`https://github.com/unitedstates/congressional-record`). In the donations database made available by OpenSecrets (`https://www.opensecrets.org/bulk-data/`), the recipients of these donations (which include both new and incumbent congressional candidates) are primarily identified via an ID which we call the 'Opensecrets ID' (*opensecrets_id*).

In order to enable research that can study associations between donations and various kinds of legislator information as well as donations and rhetoric, we map the OpenSecrets and BioGuide ID for legislators who were part of the US House over the 1995-2018 time period, using automated connectivity based on available data on legislators in both databases (such as their names) and some manual mappings in cases where automation did not find an exact match. This reliably maps the two IDs in order to enable the analyses in this work, and by making all our data publicly available, we hope that our efforts will help future research as well.

Note that we also use manually coded group assignments for PACs based on the sector, industry, and the specific category within the industry they belong to as obtained by OpenSecrets (`https://www.opensecrets.org/PACs/list.php`). This is a hierarchical coding scheme that provides for each PAC the very broad sector (such as transportation) it belongs to, its industry within that sector (such as air transport), and its specific category within that industry (such as air transport manufacturing) – providing information about the PAC on different levels of detail. This helps us investigate the association between what issues legislators choose to focus on and the donor industry they are getting donations from. Further, this helped experts interpret and validate any connection between a policy issue and a PAC that is found in our work by looking at the industry information available for the PAC within the dataset (as opposed to having to look up and find information on a PAC in order to help understand findings, since the name of the PAC may not always be informative in terms of what the PAC is about). We note that there are other databases covering similar terrain to our own [95, *cf.*]

References

95. Bonica A. Database on Ideology, Money in Politics, and Elections (DIME); 2015. Available from: `https://doi.org/10.7910/DVN/O5PX0B`.
