# Supplementary material for: Donor activity is associated with US legislators’ attention to political issues
Source: PLoS One. 2023 Sep 20;18(9):e0291169. doi: 10.1371/journal.pone.0291169 (PMC10511130; doi:10.1371/journal.pone.0291169)
Supplement: S2 Appendix — (PDF) [file pone.0291169.s002.pdf]

S2 Appendix.

Data processing.

We use the speeches, metadata, and donation information for the members of the US House over the period from 1995 to 2018, covering twelve congressional cycles. We retain representatives who gave at least fifty floor speeches and gave at least one speech in six out of the twenty-four years covered in our dataset. We retain donors (PACs) who donated at least $ 1000 to at least five of the selected representatives in at least four out of the twelve congressional cycles in our data. The final data statistics used in our machine learning model (trained over the entire dataset) are provided in S1 Table.

We use similar filtering when the models are trained separately for each congressional cycle — legislators giving at least five speeches and being recipients of at least three different donation transactions (in a particular congressional cycle) are retained for that cycle. Donors making at least three different contributions in that cycle are retained for that cycle. The final statistics for each cycle are presented in S2 Table.
