# Supplementary material for: Donor activity is associated with US legislators’ attention to political issues
Source: PLoS One. 2023 Sep 20;18(9):e0291169. doi: 10.1371/journal.pone.0291169 (PMC10511130; doi:10.1371/journal.pone.0291169)
Supplement: S3 Appendix — (PDF) [file pone.0291169.s003.pdf]

S3 Appendix.

Distribution of donations across legislators. S2 Fig and S3 Fig provide insight into our processed data and show that most PACs donate to less than a third of the legislators, and therefore, they are at least somewhat targeted), and in addition, most PACs in our data are not donating in an ideological or clearly partisan fashion, nominally donating to a mix of Democratic and Republican legislators.
