# Supplementary material for: Donor activity is associated with US legislators’ attention to political issues
Source: PLoS One. 2023 Sep 20;18(9):e0291169. doi: 10.1371/journal.pone.0291169 (PMC10511130; doi:10.1371/journal.pone.0291169)
Supplement: S4 Appendix — (PDF) [file pone.0291169.s004.pdf]

S4 Appendix.

Human issue-PAC association ratings task.

For extracting the topic-PAC associations validated by humans, we use the trained multinomial regularized logistic regression model's weights (β_1 in Eq 1) and sort those learned weight values to identify the top PACs associated with each topic.

The expert judgment or annotation task is conducted using a two-step process:

1. Two experts (political scientists) independently provide labels for each topic based on looking at its most probable words as well as its most probable documents (the full protocol and instruction set will be released). Examples of what the topic labels provided by each expert look like (for the corresponding top words) are shown in S3 Table. Note again that the top documents (based on the document-topic distribution given by the topic model) are also used to come up with the labels.

2. Using their own topic label and the top 10 PACs found by the model (examples for certain issues shown in S3 Table), the experts independently rate the PACs on a $1-3$ Likert scale. Sample of the given instructions (more instructions and recommendations/suggestions were provided as included in our data and code repository):

   "For a topic specified by using the topic label assigned in the topic labeling task from before, rate each PAC as being related to the topic (3), or not related to the topic (1) and only in cases where there seems to be some remote possibility of connection so that related or not-related is not clear, assign a (2) rating. PACs are related to a topic if they are a PAC representing an organization concerned with that same issue.
   For example, I'd rate the American College of Radiology PAC as related when the topic is Health Care (so a rating of 3), but Trinity Industries PAC (transportation industry) for that same topic would be considered not related (rating of 1)."

Note that the second step was performed for coherent and substantive issue-based topics alone. **Topics assigned the following labels in step one were not considered: Procedural/Non-Policy, In memoriam, Misc, Regulatory reform, Oversight, Political/non-policy, Regulatory, Constitutionality, Ethics.**

A snapshot of what the final annotation sheet looked like to an expert rating a topic-PAC association is given in S11 Fig.

However, in order to use the expert ratings to judge whether the model is learning something meaningful with respect to the issue-PAC associations captured in the regression weights, we need a baseline. Thus, we created another annotation task in which we randomly assigned 10 (out of the total 1002) PACs to each topic and repeat the two-step process detailed above with the same experts. The experts are simply told that these are PAC assignments for the topics from another model (and thus they do not know that these are random assignments a-priori which could have biased the annotations). We used these annotations to compare and establish that the model is indeed finding meaningful associations, and the way the learned weights rank the PACs for a topic is significantly more meaningful to domain experts than if the PACs were chosen randomly (see Fig 3 and S12 Fig).

Per the Mann-Whitney U test [61] as well as the Wilcoxon signed-rank test [62,63] for comparing expert ratings for top 10 PACs per our model versus randomly selected

10 PACs, for both experts, the model-selected top PACs are rated significantly higher than the randomly-selected PACs ($N = 400$, $p < 0.00001$). These findings clearly show that the regression model is learning meaningful topic-PAC associations, and apart from better quantitative predictive performance as compared to baselines such as having the party or state information, our model can also help get which PACs are associated with which particular issues.

Inter-expert agreement. Both experts judged issue-PAC associations for issues that they independently deemed substantive (policy-related and coherent topics) per the prior independent exercise of creating labels for topics (topic curation). Out of 60 topics, there were 14 that both experts agreed upon as either incoherent or non-substantive, while there were 3 topics for either expert that were deemed substantive by one but not the other expert. For the 40 issues deemed substantive and coherent by both experts, we compared the association scores per expert judgment for the 10 PACs provided for each issue. This resulted in a comparison of 400 scores on 1-3 Likert Scale.

We used Cohen's Kappa [64-66] to computer the inter-expert or inter-annotator agreement for the 400 instances that can be compared. Cohen's Kappa ranges from -1 to 1, with < 0 score implying less-than-chance or no agreement; a score x where $0 < x < 0.2$ implies slight agreement, $0.2 < x < 0.4$ implies fair agreement, $0.4 < x < 0.6$ implies moderate agreement, $0.6 < x < 0.8$ implies strong or substantial agreement, and $0.8 < x < 1.0$ implies nearly perfect agreement [65].

In the scenario where the PACs shown were the top 10 PACs per the machine learning model, Cohen's Kappa agreement score is 0.515 implying a moderate agreement between the two experts. In the scenario where PACs provided were selected randomly, the agreement between the experts is lower (0.339).

References

61. Mann HB, Whitney DR. On a Test of Whether one of Two Random Variables is Stochastically Larger than the Other. The Annals of Mathematical Statistics. 1947;18(1):50 – 60. doi:10.1214/aoms/1177730491.

62. Wilcoxon F. Individual comparisons by ranking methods. In: Breakthroughs in statistics. Springer Series in Statistics. Springer, New York, NY.; 1992. p. 196–202. Available from: `https://doi.org/10.1007/978-1-4612-4380-9_16`.

63. Woolson RF. Wilcoxon signed-rank test. Wiley encyclopedia of clinical trials. 2007; p. 1–3.

64. Cohen J. A coefficient of agreement for nominal scales. Educational and psychological measurement. 1960;20(1):37–46.

65. Viera AJ, Garrett JM, et al. Understanding interobserver agreement: the kappa statistic. Fam med. 2005;37(5):360–363.

66. Artstein R, Poesio M. Inter-coder agreement for computational linguistics. Computational linguistics. 2008;34(4):555–596.
