# Supplementary material for: Donor activity is associated with US legislators’ attention to political issues
Source: PLoS One. 2023 Sep 20;18(9):e0291169. doi: 10.1371/journal.pone.0291169 (PMC10511130; doi:10.1371/journal.pone.0291169)
Supplement: S5 Appendix — (PDF) [file pone.0291169.s005.pdf]

S5 Appendix.

Results with issue-attention derived from substantive policy issues alone.

Based on labels provided by experts, we remove certain non-meaningful and non-policy related topics and retrain the machine learning model. Topics assigned the following labels by any one of the two experts are removed: Procedural/Non-Policy, In memoriam, Misc, Regulatory reform, Oversight, Political/non-policy, Regulatory, Constitutionality, Ethics.

The results shown in S4 Fig confirm the same pattern that was found using all topics: *PAC* offers the most explanation for legislators' issue-attention.
