# Supplementary material for: Donor activity is associated with US legislators’ attention to political issues
Source: PLoS One. 2023 Sep 20;18(9):e0291169. doi: 10.1371/journal.pone.0291169 (PMC10511130; doi:10.1371/journal.pone.0291169)
Supplement: S6 Appendix — (PDF) [file pone.0291169.s006.pdf]

S6 Appendix.

Training details.

To compare legislator attributes in terms of their association with issue-attention in floor speeches, we train our multinomial regularized logistic regression model on some legislators to predict their averaged topic proportions, and then check the learned predictive capacity on a held-out set of legislators. Specifically, we use 50-fold cross validation (which means 2% of legislators are randomly selected to be in the held-out set for predicting their topic distribution while the model trains on the other 98% legislators to closely approximate their given topic distribution; and this is repeated 50 times). This kind of predictive framework tests the various ways to represent legislators for their capacity to generalize, i.e., given a new set of legislators and their donor, party, state, or committee information, we test how well the topic distribution predicted from that information matches the actual topic distribution for those legislators. The multinomial regularized logistic regression model is trained using the cross-entropy loss which compares the predicted and the target distributions, where the target distribution is the actual topic distribution for each legislator. When training a model for one particular congressional cycle at a time, the process is the same, except a 30-fold cross validation procedure is used to account for the lower number of legislators.

We use one particular randomly selected fold or training-validation split of the data to first tune various hyperparameters of the logistic regression model in order to select the best settings for each of the speaker models. Specifically, we allow the optimal learning rate to be selected from $\{0.000001, 0.000005, 0.00001, 0.00005\}$ and the weight decay (constant for $L-2$ regularization) to be selected from $\{0.0, 0.001, 0.005, 0.01\}$. We set number of training epochs to be $10,000$, and tune whether to enable early stopping (training to stop if the cross entropy loss does not reduce for a certain number of epochs instead of training for the full set of epochs) or not for each of the model. $L-2$ regularization and early stopping help prevent the model from overfitting on the training set.

The selected settings for each of the speaker models are provided in S4 Table. The results of the 50-fold cross validation can then be compared across the legislator attributes (results are provided in Fig 2A).

In addition to the above (one topic model and regression for the entire data spanning twelve congressional cycles), we train separate topic models (to get topic distribution for speakers) and separate regression models to predict topic distributions for each congressional cycle in our data. This helps understand the trends, such as the comparative predictive power of the topic distribution for speakers (US House representatives) offered by our legislator attributes, change over time.

The process is the same as described above except the data on floor speeches and donations used is for a particular 2-year congressional cycle at a time. Number of topics (K) for topic modeling over speeches in each cycle is set to 30. The resulting number of speakers and number of different features are shown in S2 Table. We then train logistic regression models for each cycle (this time using 30-fold cross validation), with results shown in Fig 2B.
