# Supplementary material for: Donor activity is associated with US legislators’ attention to political issues
Source: PLoS One. 2023 Sep 20;18(9):e0291169. doi: 10.1371/journal.pone.0291169 (PMC10511130; doi:10.1371/journal.pone.0291169)
Supplement: S7 Appendix — (PDF) [file pone.0291169.s007.pdf]

S7 Appendix.

Statistical testing.

For the model trained on the entire dataset, using the results of the 50-fold cross validation, we compared the 50 validation set results for the *PAC* attribute with all the other attributes, and found that *PAC* exhibits significantly ($N = 50$, $p < 0.05$) more predictive value than all the other attributes (compared pairwise) using the non-parametric Mann-Whitney U statistical test [61].

As shown clearly in the main set of results in Fig 2, the top two attributes in terms of their predictive capacity are *PAC* and *Committee*. For models trained separately on individual congressional sessions or cycles, we compare each of these two attributes to every other attribute using the 30-fold cross-validation results. The comparisons for *PAC* are shown in S5 Table and the comparisons for *Committee* are shown in S6 Table — *PAC* is significantly more predictive than *Committee* in seven out of twelve congressional cycles, and ten out of twelve congressional cycles when compared with any other attribute. We also find that *PAC* and *Committee* information potentially contain complementary knowledge, as the results improve when these two attributes are combined (S8 Fig and S9 Fig).

References

61. Mann HB, Whitney DR. On a Test of Whether one of Two Random Variables is Stochastically Larger than the Other. The Annals of Mathematical Statistics. 1947;18(1):50 – 60. doi:10.1214/aoms/1177730491.
