# Supplementary material for: Donor activity is associated with US legislators’ attention to political issues
Source: PLoS One. 2023 Sep 20;18(9):e0291169. doi: 10.1371/journal.pone.0291169 (PMC10511130; doi:10.1371/journal.pone.0291169)
Supplement: S8 Appendix — (PDF) [file pone.0291169.s008.pdf]

S8 Appendix.

***PAC* versus *Committee* legislator attributes.**

We compare *PAC* and *Committee* legislator attributes in S8 Fig and S9 Fig — while PAC is more predictive, combining the two attributes offers more explanation for legislators' issue-attention and suggests that there is some complementary information in these two attributes or explanatory variables.
