# Supplementary material for: Donor activity is associated with US legislators’ attention to political issues
Source: PLoS One. 2023 Sep 20;18(9):e0291169. doi: 10.1371/journal.pone.0291169 (PMC10511130; doi:10.1371/journal.pone.0291169)
Supplement: S9 Appendix — (PDF) [file pone.0291169.s009.pdf]

S9 Appendix.

Results with combinations of various legislator attributes.

We concatenate non-donor representations or attributes of legislators (*Committee*, *State*, and *Party*) with the *PAC* attribute to study if there is complementary information available in these attributes. S11 Fig shows the held-out set results for the model trained on the entire dataset. We find that in most cases, no additional predictive or explanatory value is derived over what is offered by *PAC*, with the combination with *Committee* being the notable examination as discussed above.
