# Supplementary material for: Donor activity is associated with US legislators’ attention to political issues
Source: PLoS One. 2023 Sep 20;18(9):e0291169. doi: 10.1371/journal.pone.0291169 (PMC10511130; doi:10.1371/journal.pone.0291169)
Supplement: S10 Appendix — (PDF) [file pone.0291169.s010.pdf]

S10 Appendix.

Procedure for surfacing potential temporal connections.

Our framework, including topic modeling and multinomial regularized logistic regression, was both used on the entire data used all at once (the 'overall' model), and also used for each individual congressional cycle separately. Since we want to uncover potential connections between donation and speech events that occur in proximity to one another, we use the topics uncovered in a particular cycle in order to use the more fine-grained modeling as the starting point. For each congressional cycle, we use that cycle's regression model weights (β_1 in Eq (1)) to identify the top topics for a PAC. We match a particular cycle's topic modeling outputs with the topic modeling outputs of the model run on the entire data of floor speeches by using the top terms in the topic-word distributions: if at least five out of the top twenty terms for the topics are the same, they are considered a match. Future work should ideally get labels and issue-PAC relevance judgments for every congressional cycle, but since that would entail the annotation procedure being carried out twelve more times, we use our topic-matching proxy in order to enable the use of the results of expert ratings for issue-PAC associations made using the multinomial regularized logistic regression model outputs trained on the entire data. A topic (in a particular congressional cycle) is considered relevant for the PAC if the issue it matched with (uncovered by the overall topic model) has that PAC *a)* among the top 10 per the overall multinomial regularized logistic regression model and *b)* was rated as relevant (3 on 3-point Likert scale) in at least one of the two independent human judgments. These relevant PAC-topic pairs for every cycle are then used as the starting points for our method outlined below as an algorithmic workflow. Our algorithm or procedure is visualized as a flowchart in S13 Fig.

Our aim here is to filter the relevant PAC-topic pairs to those that show a potential temporal connection, i.e., the PAC not only seems to be associated with the topic, but also seems to donate closer to the date a speech on that topic is made. To do so, we iterate over each topic t and PAC p in each of the relevant topic-PAC pairs, and:

1. We use the document-topic posterior learned by the topic model (trained on the particular cycle's floor speeches) to identify speeches that are 'on' the topic t (based on the topic proportion in the speech being more than uniform and the topic being in the top 3 for the speech) and also the speeches in that cycle that are not 'on' that topic t (based on the topic proportion being less than uniform and the topic ranked in the bottom half of all topics for that speech).

2. For each of these two lists of speeches (on the topic t, and not on the topic t), we take the date of each speech, and find the closest date of donation made by the PAC p to the legislator who gave that particular speech. We record that donation amount divided by the number of days between the date of that speech and the date of donation to the legislator giving that speech by PAC p — the idea being that a high donation amount by PAC p given to the legislator close to their speech on topic t is what signifies a potential temporal connection.

3. We thus have a list of values for speeches on topic t and a list of values for speeches not on that topic. We conduct a Welch t-test (which does not assume equal population variance) [67] to identify cases where the first list represents a distribution of values that is significantly greater than the second list ($p < 0.005$).

4. Based on the above significance test, we select relevant PAC-topic pairs with a potential temporal connection for the cycle. We can then look at speeches made on the topic and cases of the relevant PAC donating an amount much higher than their mean donation amount during that congressional cycle to the legislator who gave that speech within a particular time window around the speech. Some examples of such speeches and donations are shown in S7 Table.

5. Based on aggregating cases of such speeches, we can look at PAC and legislators that recur in such temporal connections, i.e., the PAC donating a larger-than-their-usual amount to a legislator giving the speech on a relevant issue, in, say, a 7-day period around the date of that speech, and see if that same PAC-legislator pair occurs multiple times either during a cycle or even across cycles. One example of such cases uncovered by our method is shown in Fig 4, and more examples of such legislator-PAC relationships we found (and obtained face-validity through straightforward search engine queries) are provided in S14 Fig and S15 Fig.

References

102. Welch BL. The generalization of 'STUDENT'S'problem when several different population variances are involved. Biometrika. 1947;34(1-2):28–35.
