# Supplementary material for: Donor activity is associated with US legislators’ attention to political issues
Source: PLoS One. 2023 Sep 20;18(9):e0291169. doi: 10.1371/journal.pone.0291169 (PMC10511130; doi:10.1371/journal.pone.0291169)
Supplement: S11 Appendix — (PDF) [file pone.0291169.s011.pdf]

S11 Appendix.

Robustness check for a potential dependency of our finding on the choice of topic modeling method used to obtain legislators' issue-attention by using three different topic models other than LDA.

In our main experiments as described in the *Materials and methods* section, we use Gibbs-sampling based LDA as the choice of topic modeling method in order to derive legislators' issue-attention. Our finding does not depend on the automated topic modeling output alone, but as discussed in S5 Appendix, is also replicated when using coherent political issues curated by experts based on the topic modeling output. However, since the posterior distribution used to instantiate issue-attention is based on topic modeling output, we also test if our finding holds across different types of topic modeling methods. We retain the exact framework and decisions described in the *Materials and methods* section, and only change the choice of topic modeling method used to obtain topic distributions (resulting in a new dependent variable or y used in the regularized multinomial logistic regression model). We then compare the four main legislator attributes — *PAC*, *Committee*, *State*, and *Party*. This robustness check is performed across three different topic modeling methods (other than LDA):

1. **Structured Topic Model (STM):** We use the STM model instead of LDA, and in order to use the same framework, do not use metadata in the model and obtain topic distributions using floor speech data. We use the STM package in R: cran.r-project.org/web/packages/stm/index.html.

2. **Non-negative Matrix Factorization (NMF):** NMF models are used for dimensionality reduction based on uncovering important latent components, lending themselves to topic modeling. We use the implementation of NMF provided in the *scikit-learn* package in Python: scikit-learn.org/stable/modules/generated/sklearn.decomposition.NMF.html.

3. **Contextualized Topic Model (CTM):** The CTM model effectively uses the strength of pre-trained large language model-based contextual embeddings in topic modeling, in an attempt to alleviate the limitations of the bag-of-words representation for the document collection typically used in topic modeling methods. Contextualized embeddings can incorporate linguistic context and make use of the sequential nature of language data. Specifically, we use the Combined Topic Model or CombinedTM approach that represents sentences or sequences of text using the Sentence BERT (SBERT) approach, as documented in the publicly available implementation of the CTM model at github.com/MilaNLProc/contextualized-topic-models.

Our main finding is replicated across all these three additional topic modeling methods: PACs are significantly more associated with legislators' issue-attention in floor speeches than other legislator attributes. Results for STM, NMF, and CTM are presented in Figs S14, S15, and S16 respectively. Using the statistical significance testing procedure described in S7 Appendix, we confirm that the *PAC* legislator attribute is a significantly better predictor of issue-attention than *Committee*, *State*, and *Party* ($N = 50, p < 0.05$) in all these three cases that use different topic modeling approaches.
