# Supplementary material for: Donor activity is associated with US legislators’ attention to political issues
Source: PLoS One. 2023 Sep 20;18(9):e0291169. doi: 10.1371/journal.pone.0291169 (PMC10511130; doi:10.1371/journal.pone.0291169)
Supplement: S12 Appendix — (PDF) [file pone.0291169.s012.pdf]

S12 Appendix.

Robustness check for a potential dependency of our finding on the order of documents used to train the LDA topic model.

For the Gibbs-sampling-based LDA topic model, the topic modeling output *might* change if the documents used to train the model are provided in a different order. We expect such changes to be minor and not have any substantial effect on the final output given our large dataset of documents. Additionally, S11 Appendix establishes that our finding does not depend on the choice of topic modeling method.

As a further assurance for the use of the LDA topic model itself for our work and the potential use of this model in other related research, we establish that our finding about donor activity's association with issue-attention is also not dependent on the order of documents in the training data when issue-attention is quantified using LDA.

We randomly shuffle the order of the speeches used to train the model ten times and reconduct our main experiment using each of these ten different LDA-based document-topic distributions (which are then aggregated to obtain legislators' issue-attention). We find that our finding holds for all of these ten different LDA runs, where the *PAC* legislator attribute remains a significantly better predictor of issue-attention than *Committee*, *State*, and *Party* ($N = 50, p < 0.05$; following the same procedure for statistical significance testing as detailed in S7 Appendix).

Further, for each of these four attributes, we obtain the mean % bit reduction scores across the ten topic modeling runs and compare these means. This comparison is presented in S16 Fig. Our results, in terms of measured explanation of issue-attention when modeled via different legislator attributes, are stable across the different runs of topic models (with different orders of training documents). Our main finding is again replicated in aggregate across these ten different runs: PACs are significantly more associated with legislators' issue-attention in floor speeches than other legislator attributes. The order in which the floor speeches are provided to our Gibbs-sampling-based LDA topic modeling method does not seem to have any bearing on our findings.
