# Supplementary material for: Donor activity is associated with US legislators’ attention to political issues
Source: PLoS One. 2023 Sep 20;18(9):e0291169. doi: 10.1371/journal.pone.0291169 (PMC10511130; doi:10.1371/journal.pone.0291169)
Supplement: S13 Appendix — (PDF) [file pone.0291169.s013.pdf]

S13 Appendix.

Robustness check for a potential dependency of our finding on the number of topics used to train the LDA topic model.

In our work, we do not only rely on the obtained topic distribution for our main finding (presented in Fig 2A), but conduct an extensive expert curation process to additionally obtain a coherent set of labels for political issues used in our analyses (step 1 in the human curation procedure discussed in S4 Appendix), and show that after removing non-coherent or non-substantive categories or issues, our main finding continues to hold (S5 Appendix and S4 Fig): PACs offer the highest association with issue-attention. Therefore, the initial number of topics (60 in our work) is reduced to a set of identified categories based on expert judgment (48 in our work), and we believe this is an important recipe that established the reliability of our results since our finding holds for both the initial set of automatically discovered topics as well as the expert-refined or curated set of issues.

Our results reported in Fig 2A use an LDA model trained using sixty topics ($K = 60$). In recognition that different practitioners might rely on a different number of topics for their initial estimates, we conduct an additional robustness check where we derive issue-attention using a different value for the number of topics or K.

In S18 Fig, S19 Fig, S20 Fig, and S21 Fig, we show our results to highlight that our main finding holds if we instead used $K = 30$, $K = 45$, $K = 90$, or $K = 120$ respectively. PAC offers a significantly higher association with issue-attention in all these cases compared with *State* and *Party*. Except for $K = 30$, PAC offers a significantly stronger association with issue-attention than *Committee* (and equivalent association in the case of $K = 30$). Significance testing follows the procedure laid out in S7 Appendix ($N = 50, p < 0.05$). We, therefore, show that our finding is robust across a range of different values for the number of topics (K).
