# Supplementary material for: Donor activity is associated with US legislators’ attention to political issues
Source: PLoS One. 2023 Sep 20;18(9):e0291169. doi: 10.1371/journal.pone.0291169 (PMC10511130; doi:10.1371/journal.pone.0291169)
Supplement: S14 Appendix — (PDF) [file pone.0291169.s014.pdf]

S14 Appendix.

Detailing and comparing different types of PACs in our processed dataset: Business PACs offer a significantly stronger association with issue-attention than labor PACs.

As noted in the Materials and methods section in the main text, we use publicly available bulk data on donation transactions as well as donors provided by OpenSecrets (https://www.opensecrets.org/bulk-data/). In this data, PACs are divided into five different types of PACs: Business, Labor, Ideological, Other, and Unknown. In our work, we only consider business and labor PACs.

In our processed dataset, we have a total of 1002 PACs (see S2 Appendix for details on data processing). Examining how this divides by the type of PAC, we find that our final set of PACs consists of 937 business PACs and 65 labor PACs. For the 758 legislators in our processed dataset across 1995-2018, business PACs donated to at least 4 legislators, to a maximum of 715 legislators, and to 251.6($\pm$126.7) on average. Labor PACs, on the other hand, donated to 27.7($\pm$19.9) legislators on average and a maximum of 61 legislators. Additionally, there were 11 labor PACs that did not donate to a single legislator in our processed dataset.

Finally, we examine the relative predictive capacity for legislators' issue-attention offered by these two types of PACs. We reconduct our main experiment outlined in the Materials and methods section in the main text, but use only a subset of PACs (based on type) to represent the individual legislators (X). S22 Fig presents our results. We find that business PACs offer a significantly higher association with issue-attention compared with labor PACs. Considering *all* PACs, however, still offers a significantly higher association than considering just business PACs, highlighting the potentially complementary nature of information offered by these two types of PACs when explaining legislators' issue-attention. Significance testing follows the procedure laid out in S7 Appendix ($N = 50, p < 0.05$).
