# Supplementary material for: Donor activity is associated with US legislators’ attention to political issues
Source: PLoS One. 2023 Sep 20;18(9):e0291169. doi: 10.1371/journal.pone.0291169 (PMC10511130; doi:10.1371/journal.pone.0291169)
Supplement: S15 Appendix — (PDF) [file pone.0291169.s015.pdf]

S15 Appendix.

Details about constructing two additional legislator attributes — *Seniority* (number of congressional terms served) and *District Marginality* (how close their district is in voting in US presidential elections) — and discussing comparison with other attributes at the level of specific congressional sessions.

We discuss the construction of two additional legislator variables — *Seniority* (number of congressional terms served) and *District Marginality* (how close a legislator's district is in voting in US presidential elections). These variables can only be constructed and compared at the level of particular congressional sessions. After defining these variables or attributes, we compare them with other attributes in order to assess the robustness of our findings about PACs' association with issue-attention in light of these new legislator attributes.

Seniority considers the number of congressional terms served by a legislator up to and including the particular Congress being considered. We obtain all possible values for this attribute for a particular Congress and use one-hot encoding as used for other attributes and discussed in the Materials and methods section. Information about the terms or congressional sessions served by legislators is available in the US Congressional Record database along other with other legislator metadata we use, such as their home state and party. This attribute can be compared with other legislator attributes across all 12 congressional sessions in our dataset.

Results presented in S23 Fig confirm that *Seniority* does not help explain or predict issue-attention, and our main finding about *PAC* being the most significant predictor of issue-attention continues to hold ($N = 30, p < 0.05$; following the same procedure for statistical significance testing as detailed in S7 Appendix).

An important consideration for possible motivating factors for US House members' behavior and actions is the district they represent, and specifically, the partisan make-up of their constituency. We use the data on presidential voting results for 2008, 2012, and 2016 US General elections at a district level provided by Daily Kos: docs.google.com/spreadsheets/d/1zLNAuRqPauss00HDz4XbTH2Hqs
CzMe0pR8QmD1K8jk8/edit#gid=0; also see
www.dailykos.com/stories/2020/11/19/1163009/-Daily-Kos-Elections-
presidential-results-by-congressional-district-for-2020-2016-and-2012.

These 2008, 2012, and 2016 presidential voting results were used to obtain the absolute difference between votes for the Democratic and Republican party candidates for the 2009-10, 2013-14, and 2015-16 congressional sessions or cycles respectively. We divided all the absolute difference values for a particular session into ten percentiles, obtaining ten data-driven levels of how marginal the district is for each session. This creates the *District Marginality* legislator attribute. We note that for a few legislators (< 10) in these sessions, district voting data was not available; we remove those legislators from consideration for all the legislator attributes we compare in order to ensure a fair, head-to-head comparison.

Results presented in S24 Fig confirm that across the three different congressional sessions, *District Marginality* does not help explain or predict issue-attention, and our main finding about *PAC* being the most significant predictor of issue-attention continues to hold ($N = 30, p < 0.05$; following the same procedure for statistical significance testing as detailed in S7 Appendix).
