# Supplementary figures and images for: Donor activity is associated with US legislators’ attention to political issues
Source: PLoS One. 2023 Sep 20;18(9):e0291169. doi: 10.1371/journal.pone.0291169 (PMC10511130; doi:10.1371/journal.pone.0291169)

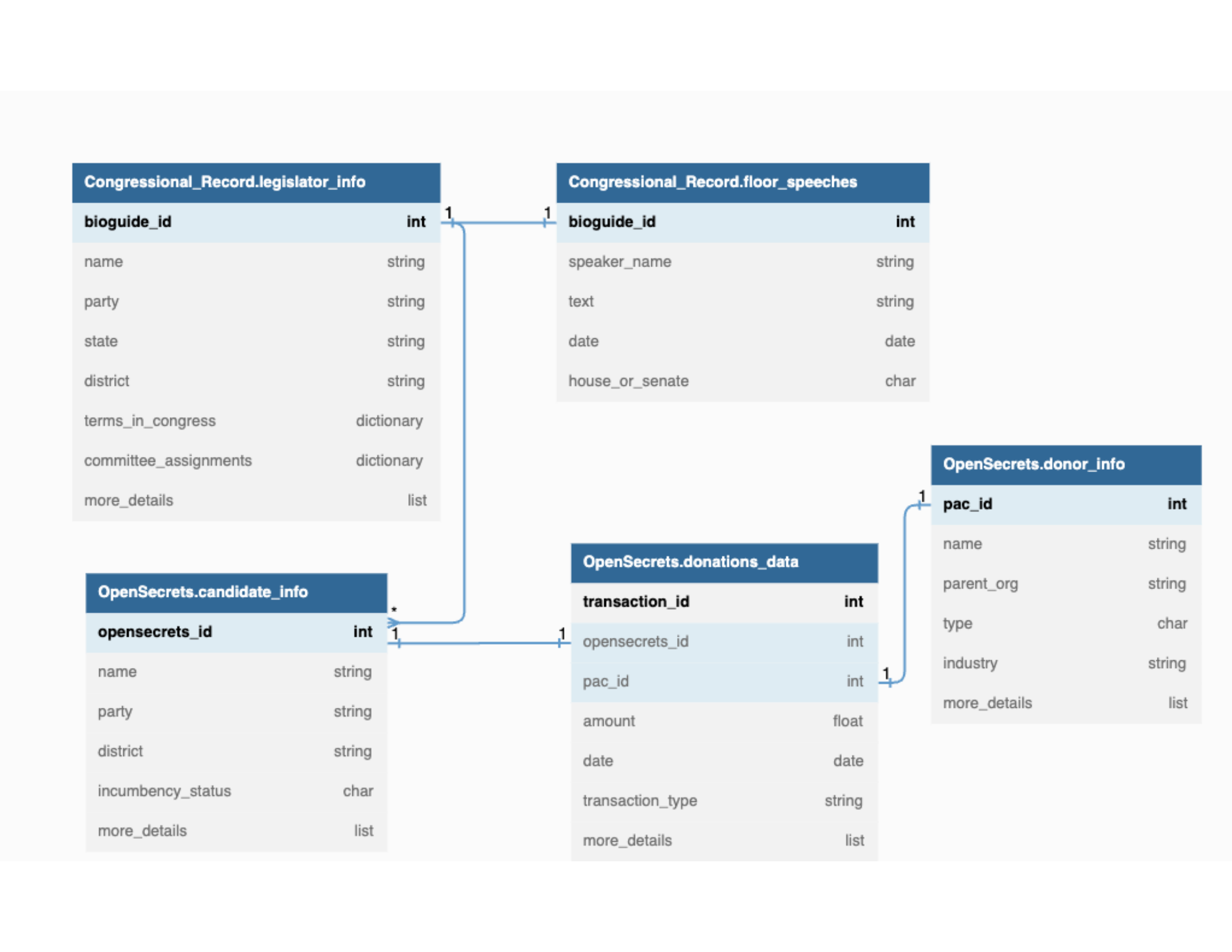

Supplement: S1 Fig — Note that not all data columns are shown for each table (relation), and the schematic aims to simply provide an overview of the structure and contents of our database. In this database we have created, the PAC contributions data runs from 1989–2018, while the floor speeches are from 1994–2020. There are 5 possible values for the type of PAC: Business, Labor, Ideological, Other, and Unknown—we only consider Business and Labor PACs in our study. (TIF) [file pone.0291169.s016.tif]

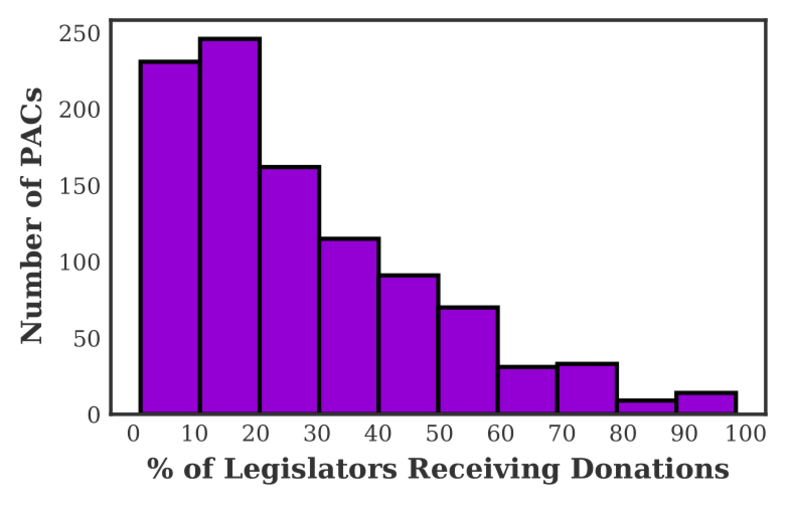

Supplement: S2 Fig — In our processed dataset used to train our machine learning framework with 758 legislators who are present and give floor speeches across 1995–2018, and 1002 PACs actively donating in the same period; most PACs tend to donate to less than a third of all the legislators they could donate to. (TIF) [file pone.0291169.s017.tif]

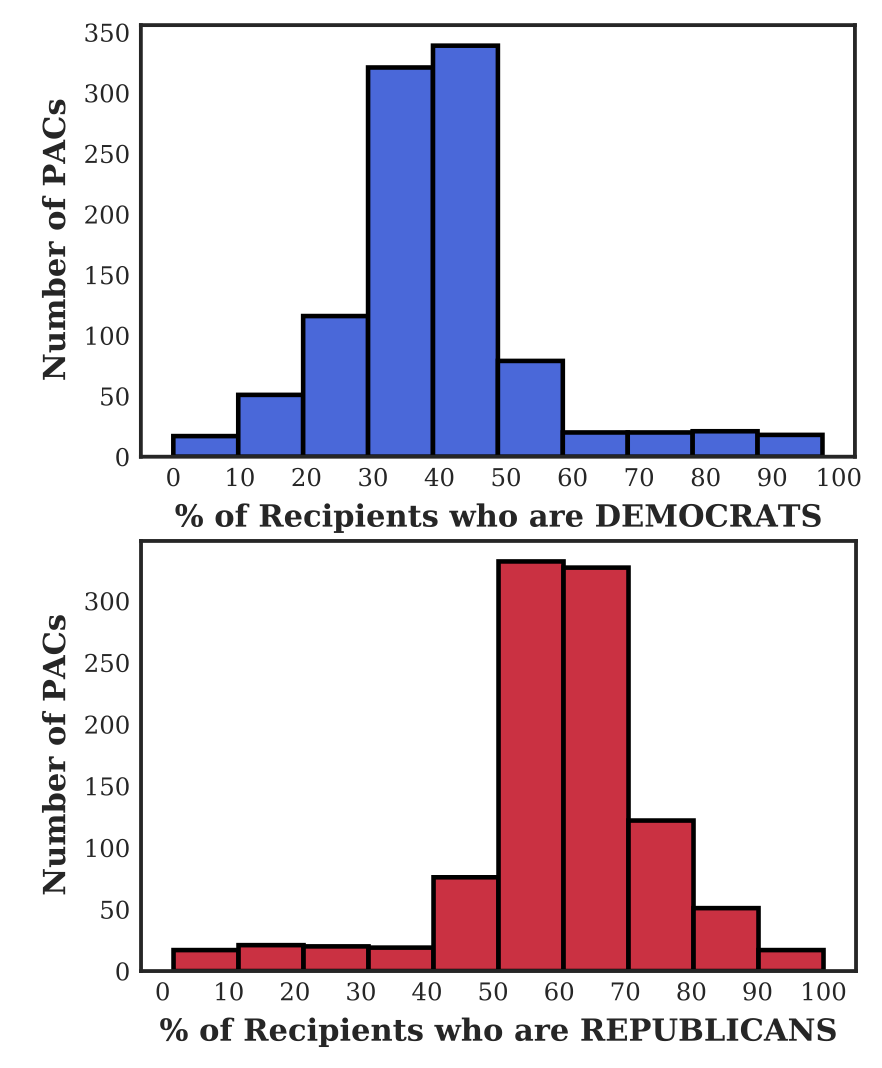

Supplement: S3 Fig — In our processed dataset used to train our machine learning framework, most PACs tend to donate across party lines instead of donating in an exclusive, partisan manner. (TIF) [file pone.0291169.s018.tif]

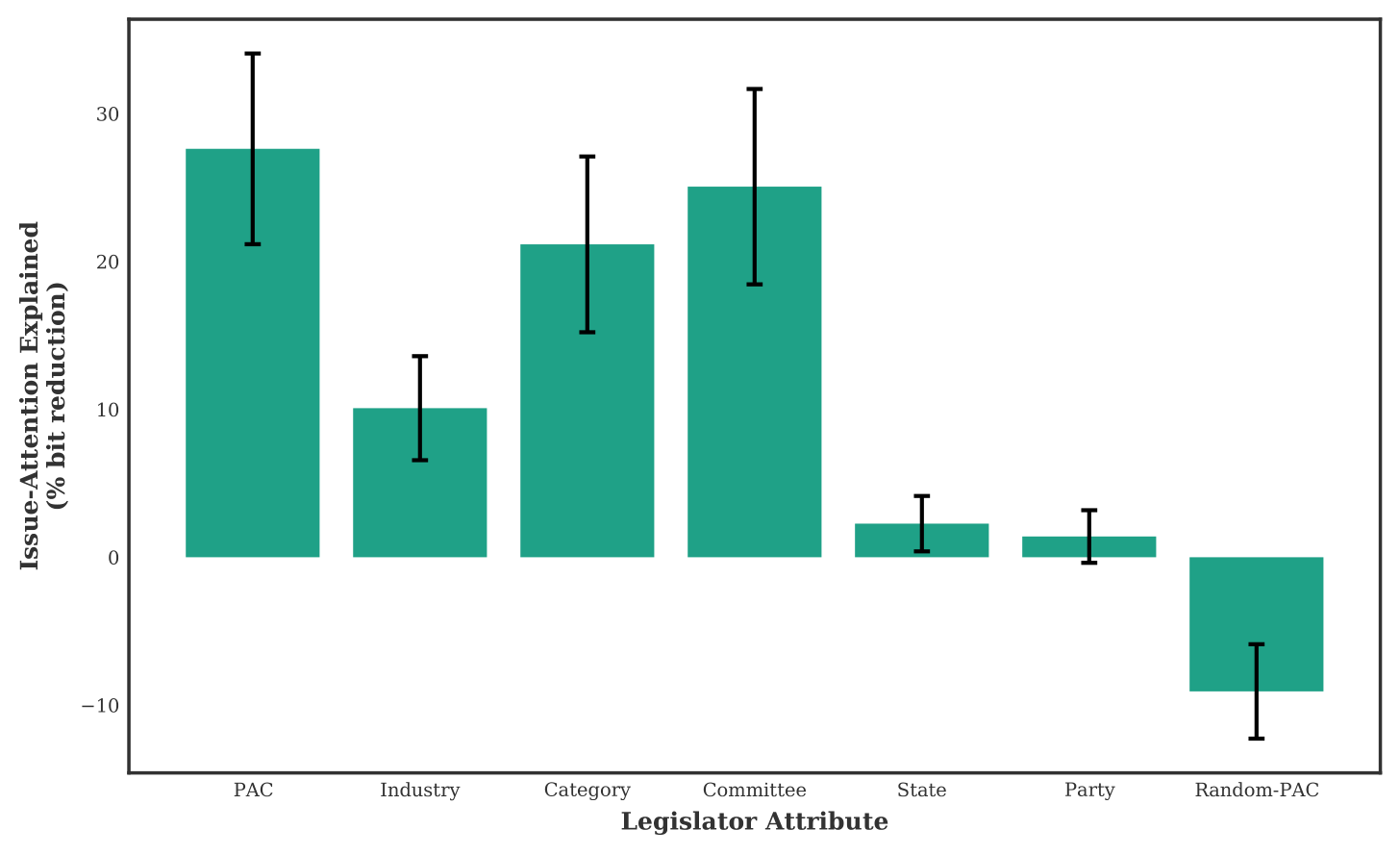

Supplement: S4 Fig — We find the same pattern as when using all topics: PAC attribute explains legislators’ issue-attention the most. (TIF) [file pone.0291169.s019.tif]

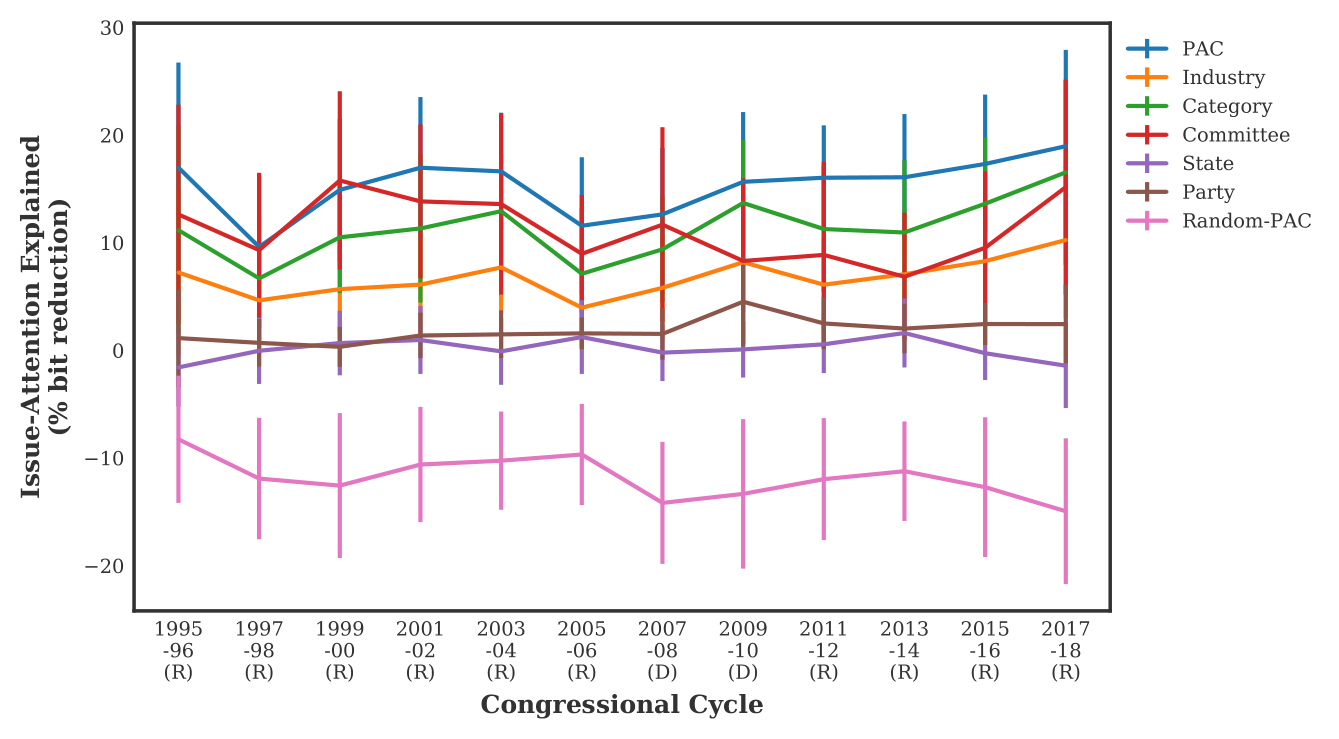

Supplement: S5 Fig — These correspond to the held-out set results (without error bars) shown in Fig 2B. (TIF) [file pone.0291169.s020.tif]

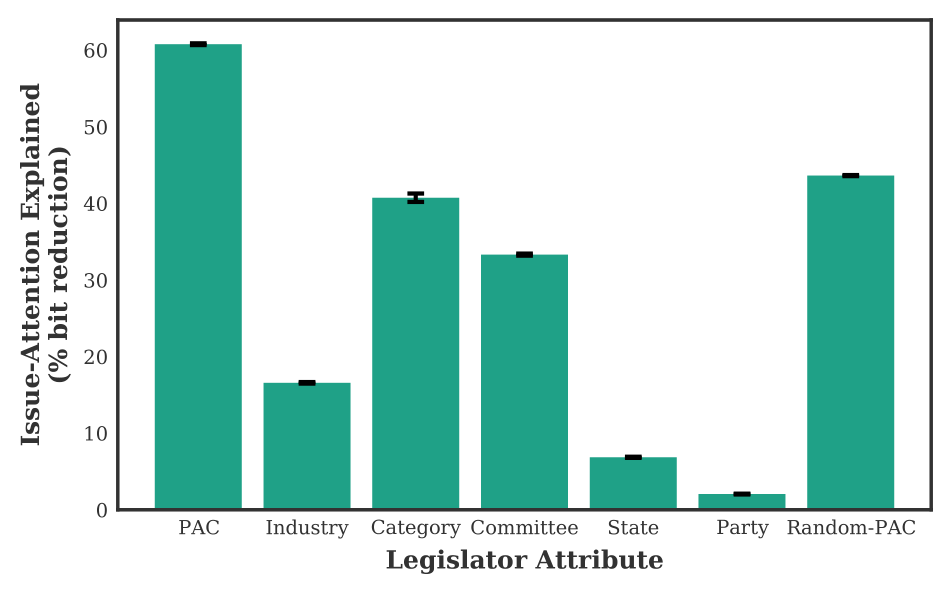

Supplement: S6 Fig — Corresponding held-out set results are shown in Fig 2A. (TIF) [file pone.0291169.s021.tif]

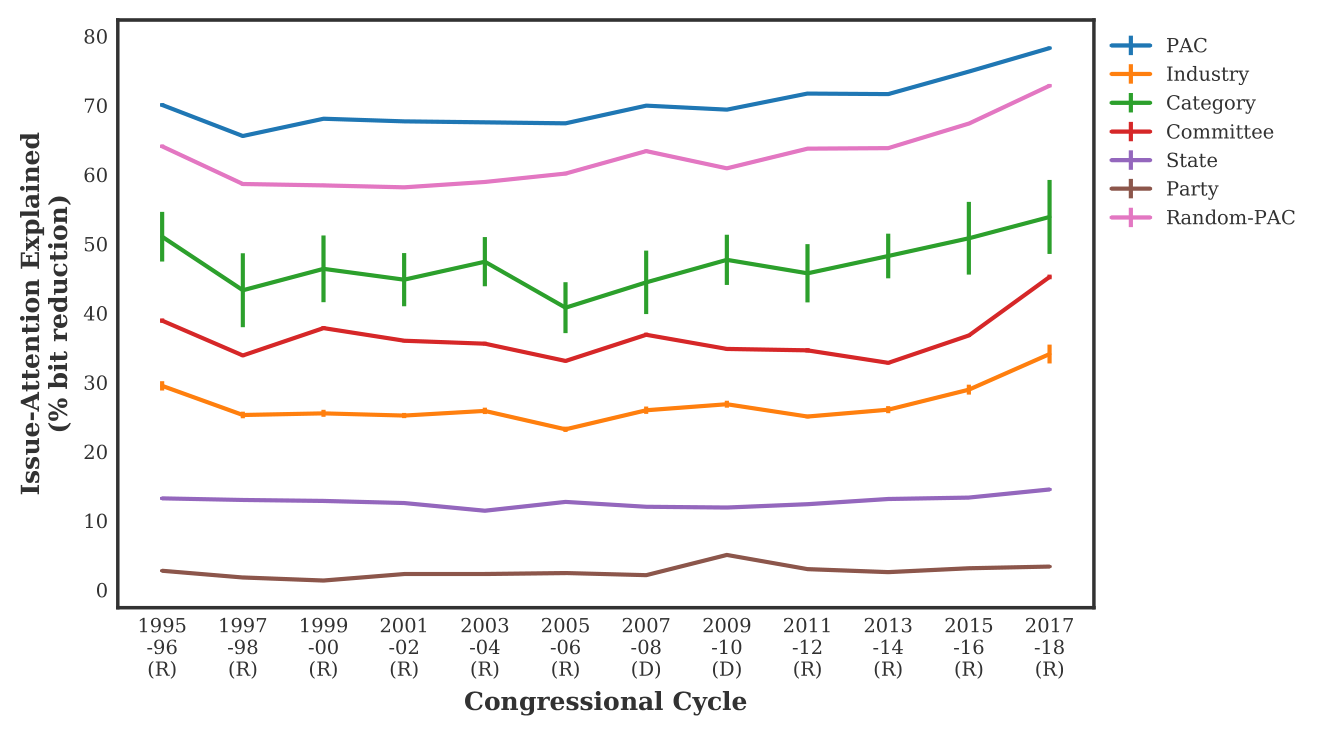

Supplement: S7 Fig — Corresponding held-out set results are shown in Fig 2B. (TIF) [file pone.0291169.s022.tif]

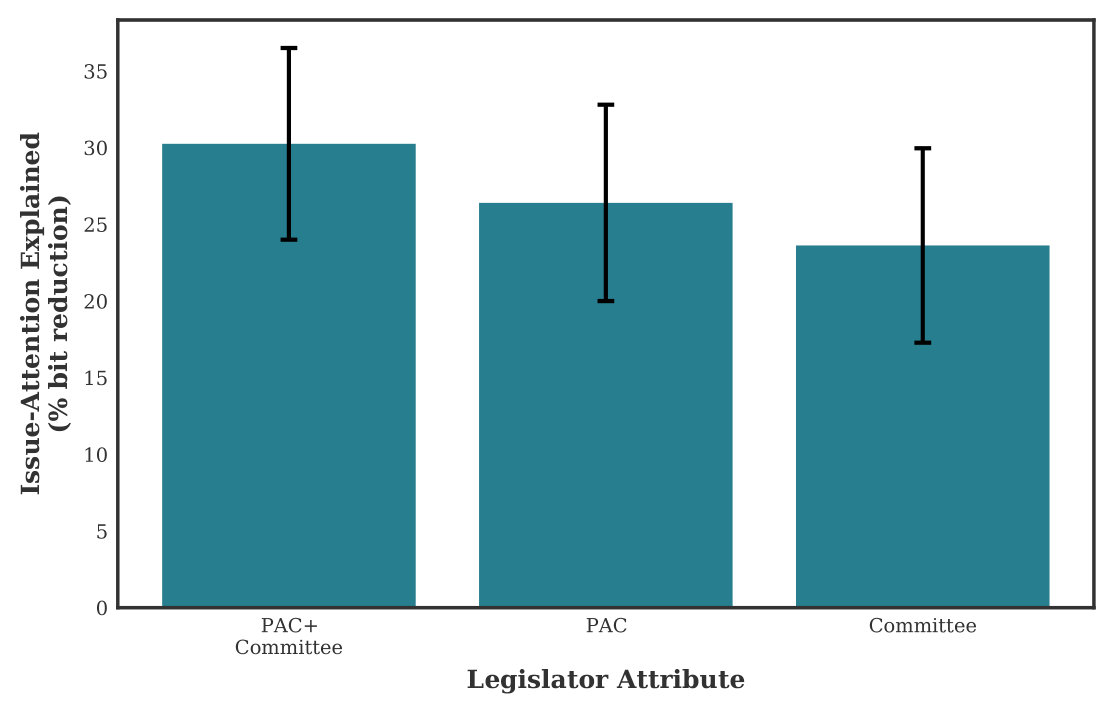

Supplement: S8 Fig — While PAC offers more association with issue-attention, results for the combined attribute set of PAC and Committee suggest some complementary information present in these two explanatory variables. (TIF) [file pone.0291169.s023.tif]

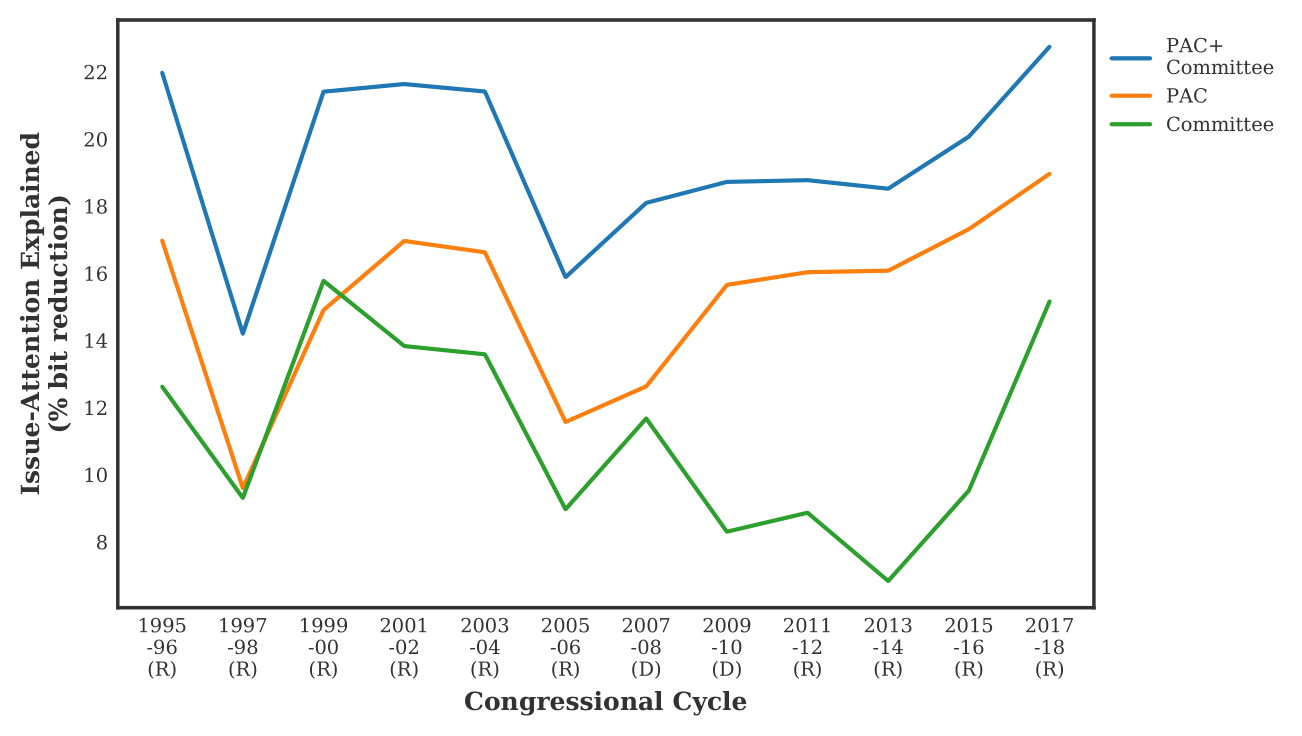

Supplement: S9 Fig — (TIF) [file pone.0291169.s024.tif]

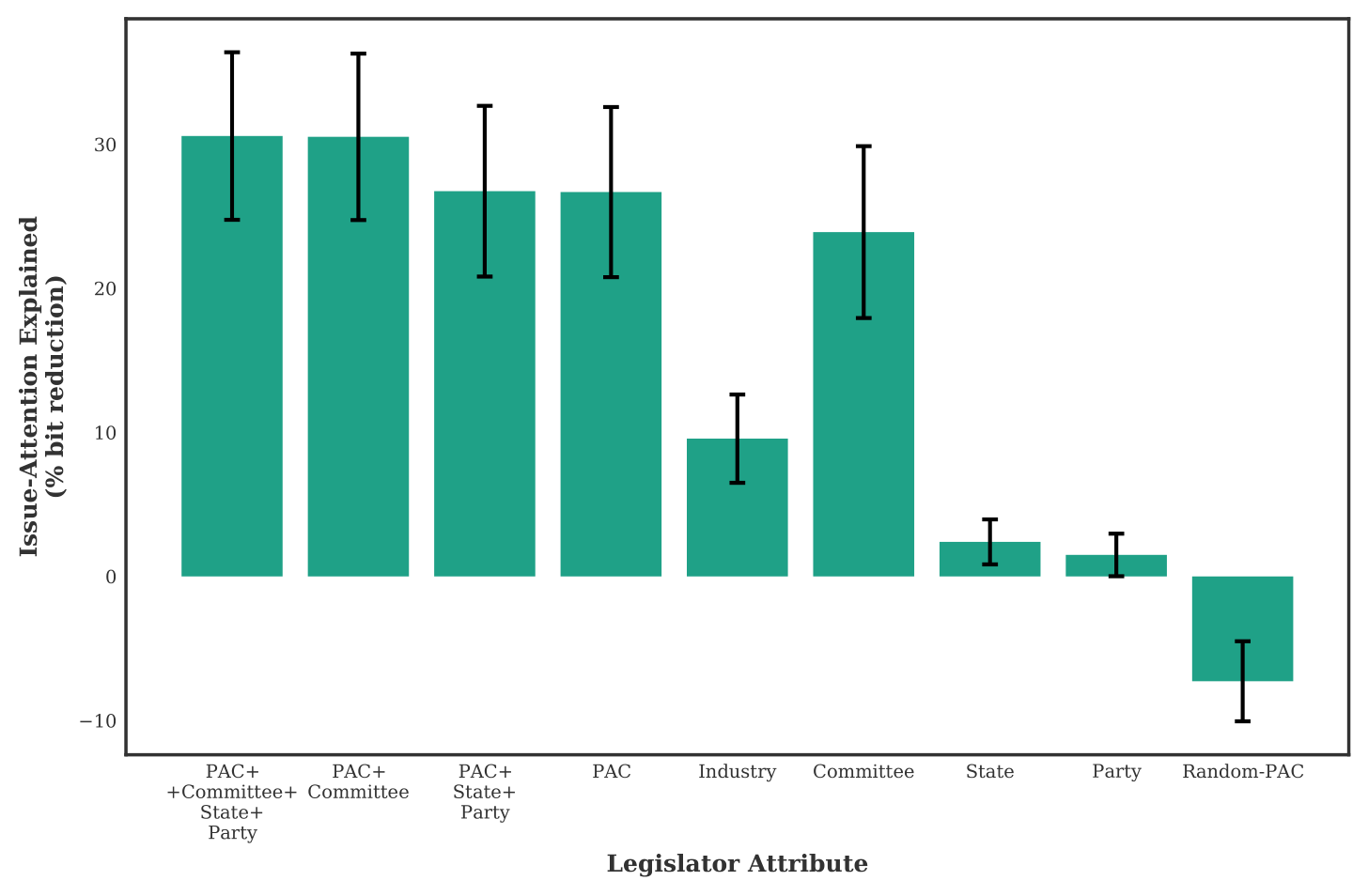

Supplement: S10 Fig — Only Committee information increases the explanatory power of the PAC attribute. (TIF) [file pone.0291169.s025.tif]

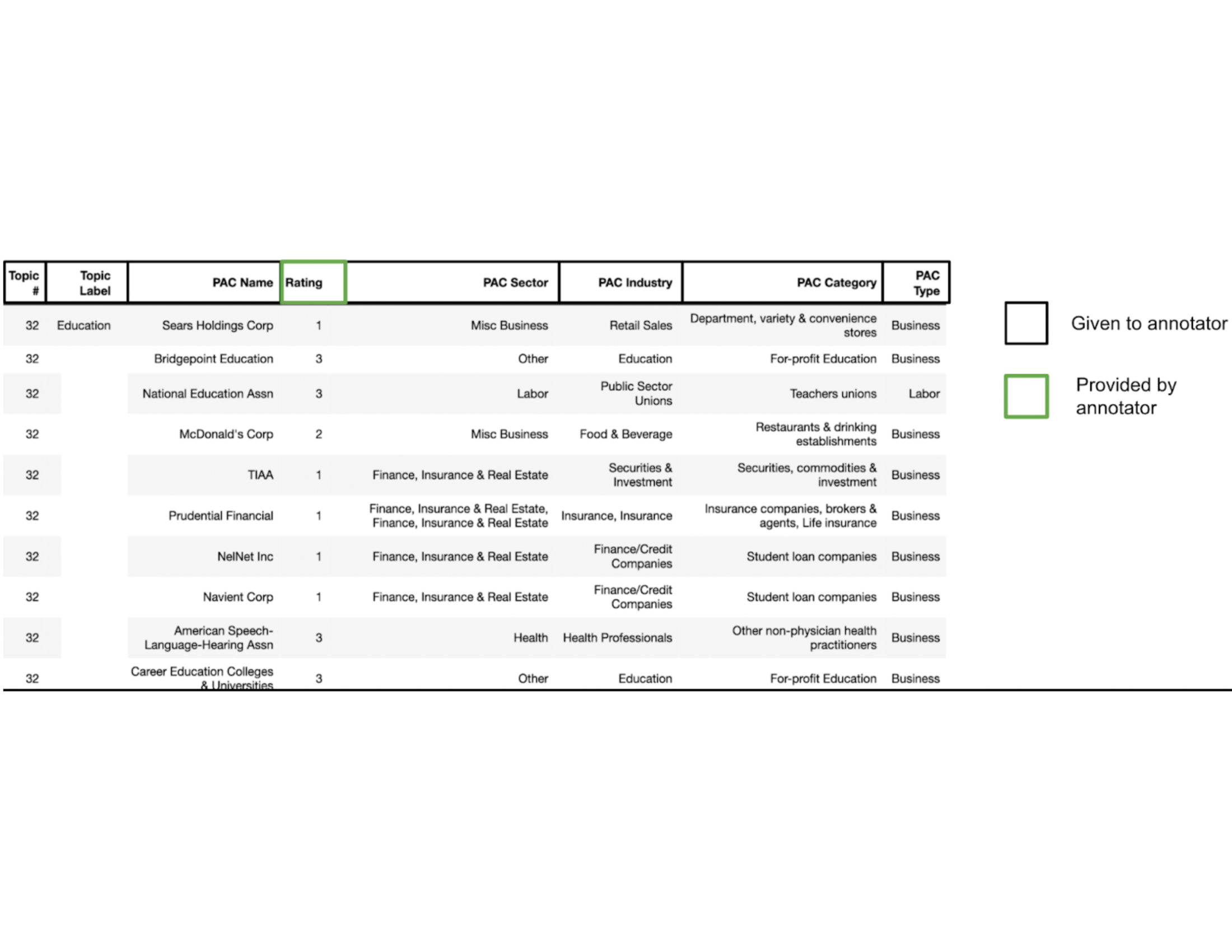

Supplement: S11 Fig — Given their own topic label, and 10 PACs, an expert rated the association of the PAC with the topic on a 1−3 Likert scale. (TIF) [file pone.0291169.s026.tif]

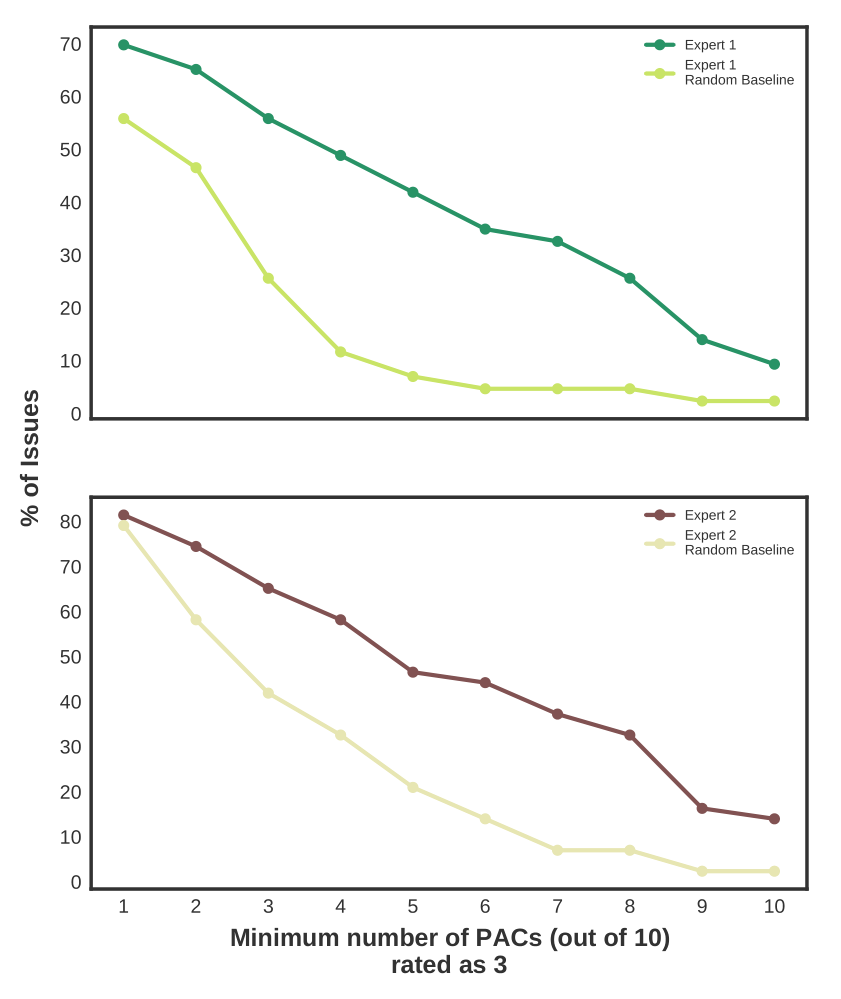

Supplement: S12 Fig — Additional results of experts rating issue-PAC associations, showing that the top PACs per issue as per our regression model’s weights are meaningful, since the model’s top 10 PACs are rated consistently higher in terms of association with the issue as compared with a random selection of 10 PACs for the issues. A value of 5 (as an example) on the x-axis here means at least 5 out of the 10 PACs shown were rated as clearly associated with the issue (a rating of 3). More PACs were rated as related to the issue, out of the 10 shown, when the PACs shown were selected based on the associations learned by our model compared with a random selection. (TIF) [file pone.0291169.s027.tif]

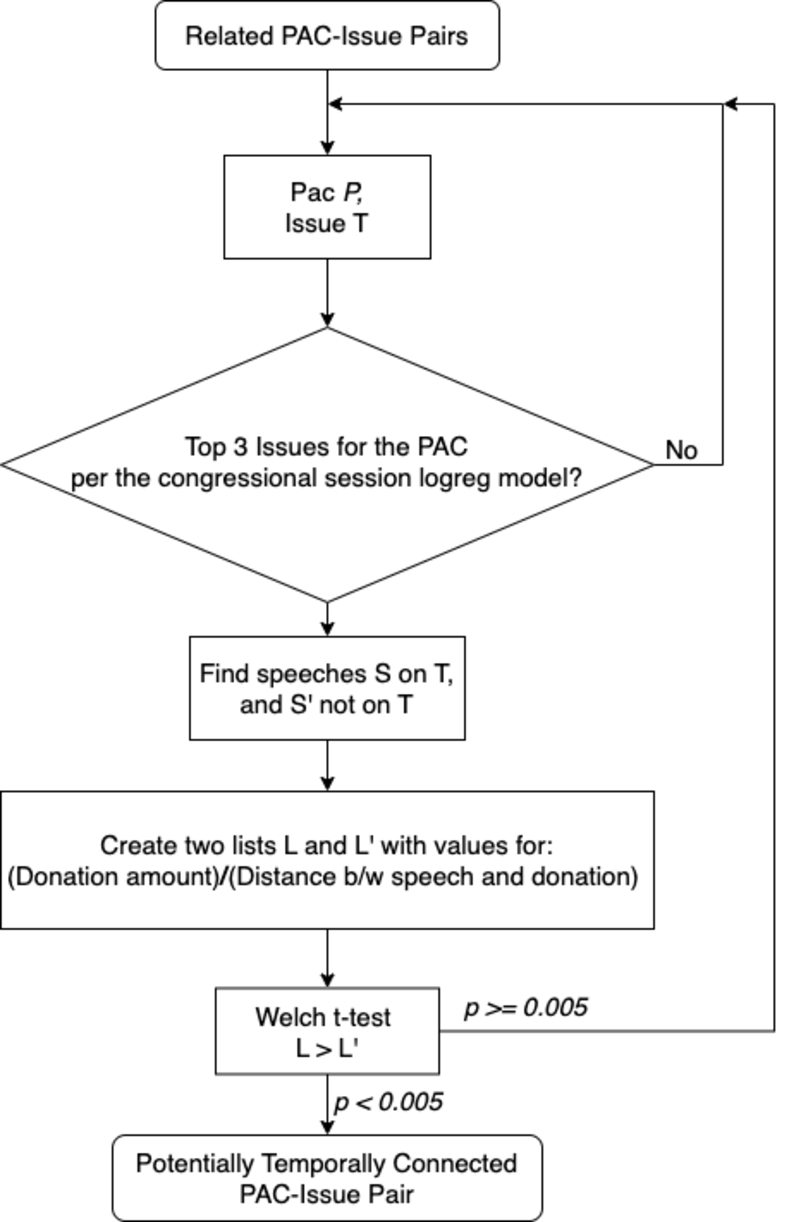

Supplement: S13 Fig — These are cases where, within a short time window (proximal), a donation made by a PAC interested in a particular issue and a speech made by the recipient on that issue co-occur. (TIF) [file pone.0291169.s028.tif]

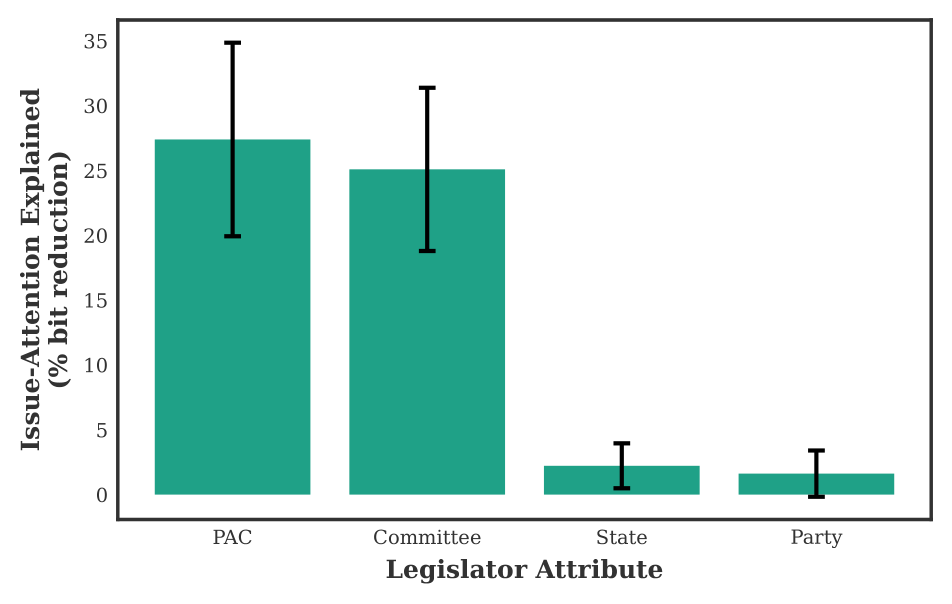

Supplement: S14 Fig — (TIF) [file pone.0291169.s029.tif]

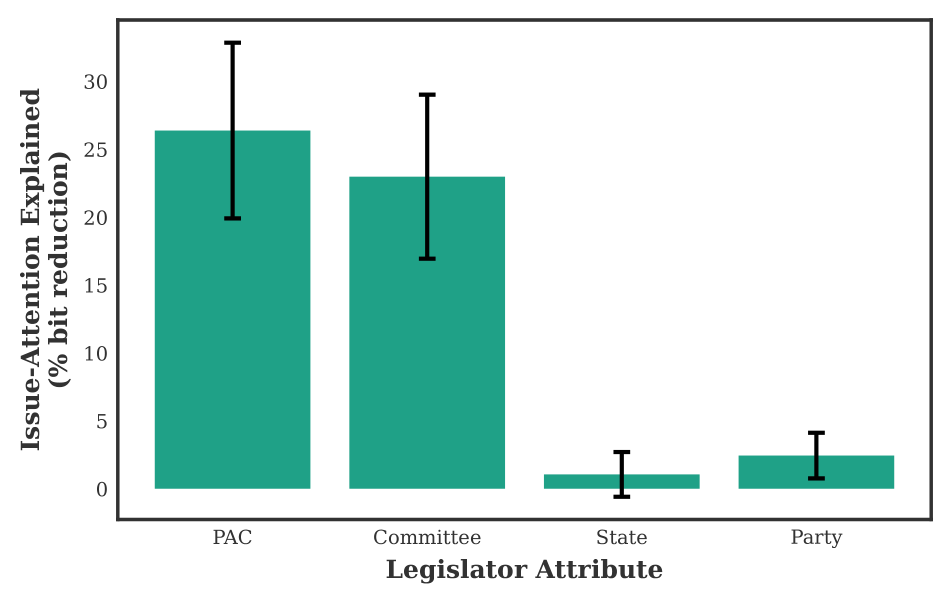

Supplement: S15 Fig — (TIF) [file pone.0291169.s030.tif]

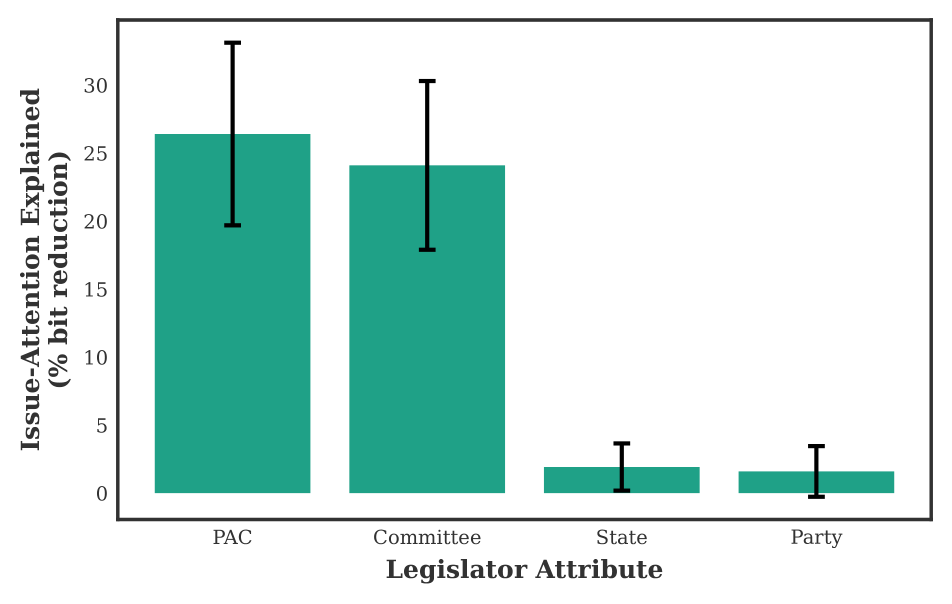

Supplement: S16 Fig — (TIF) [file pone.0291169.s031.tif]

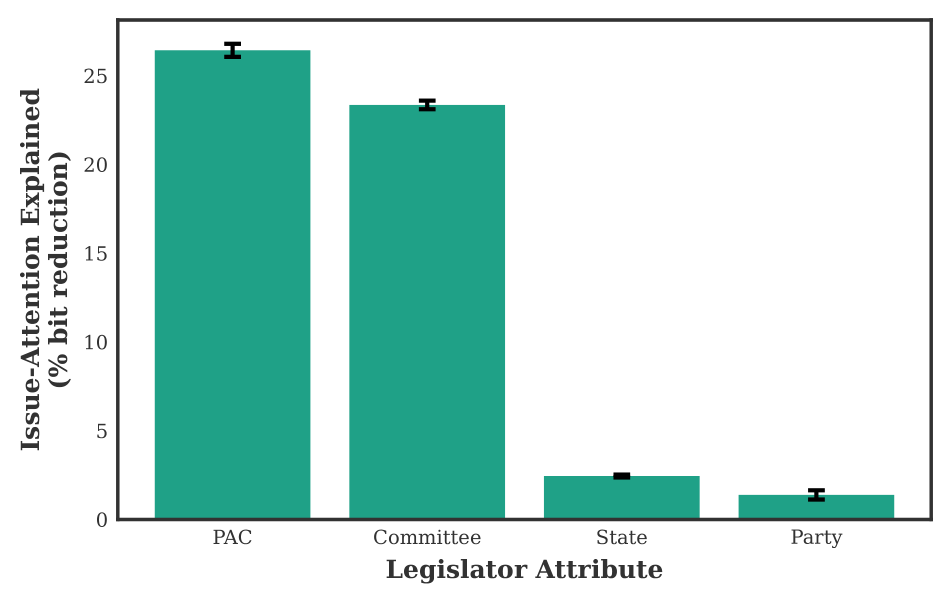

Supplement: S17 Fig — (TIF) [file pone.0291169.s032.tif]

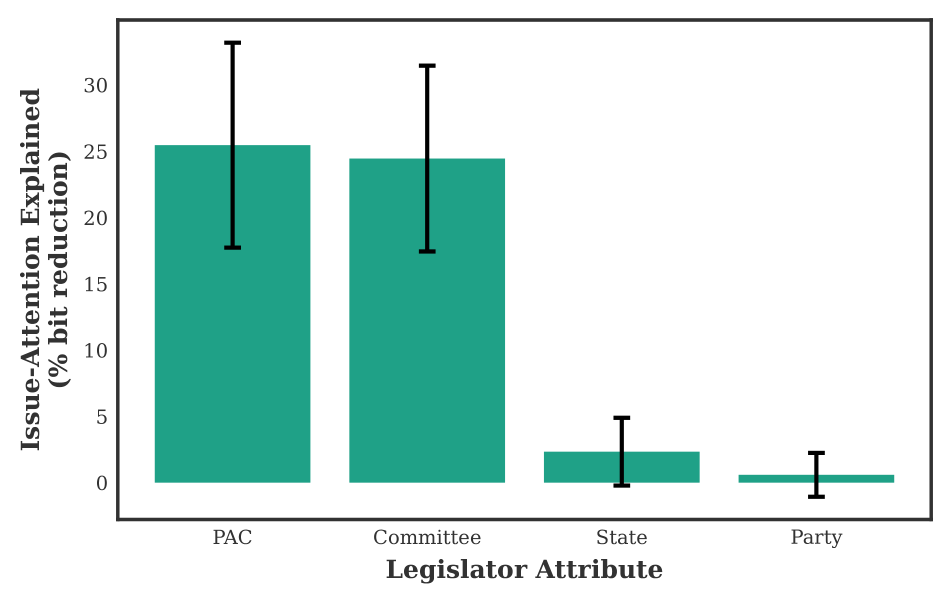

Supplement: S18 Fig — (TIF) [file pone.0291169.s033.tif]

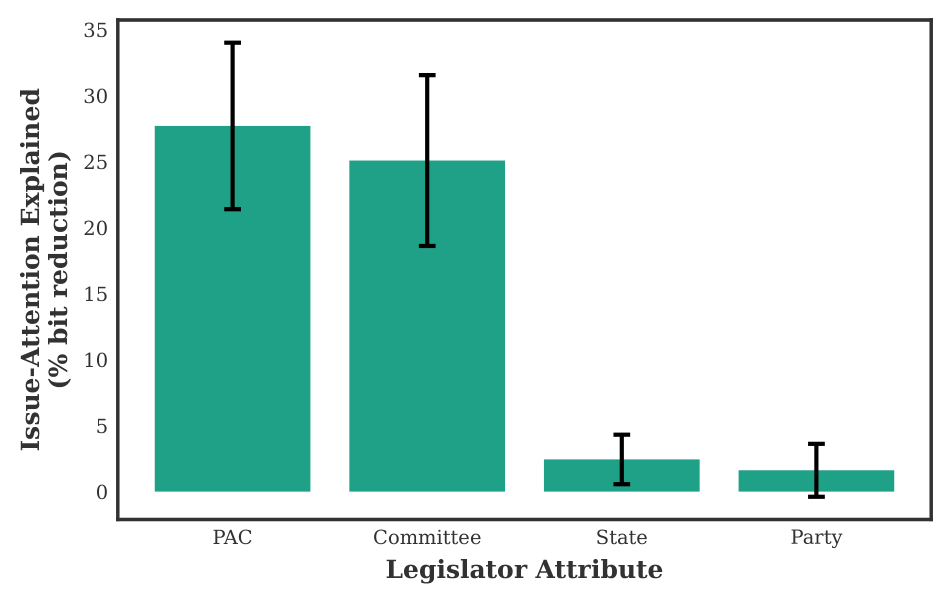

Supplement: S19 Fig — (TIF) [file pone.0291169.s034.tif]

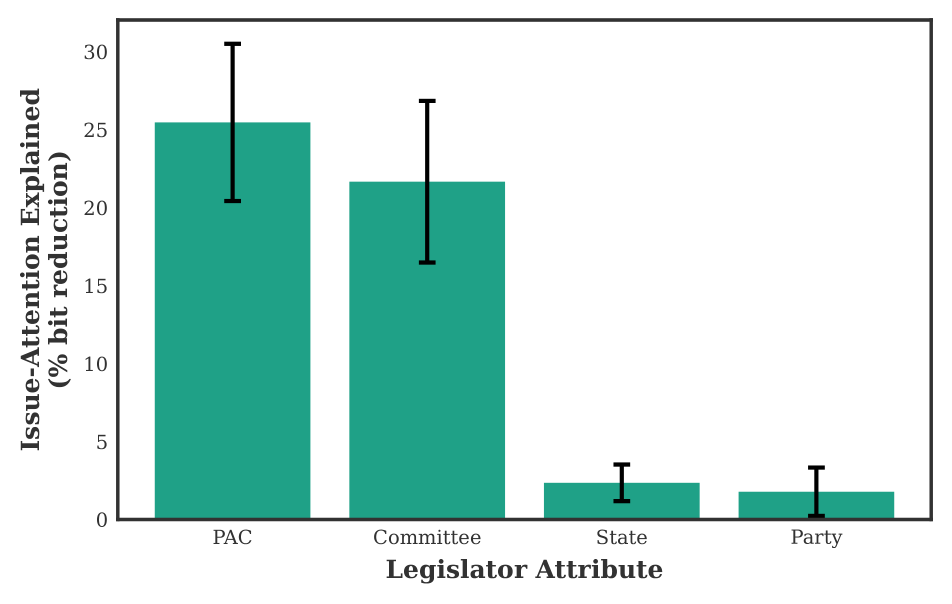

Supplement: S20 Fig — (TIF) [file pone.0291169.s035.tif]

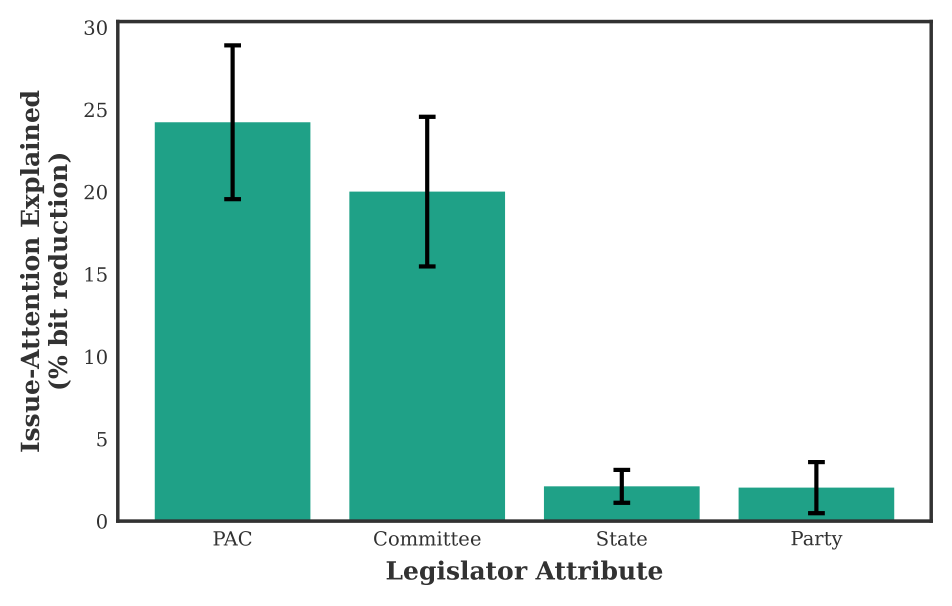

Supplement: S21 Fig — (TIF) [file pone.0291169.s036.tif]

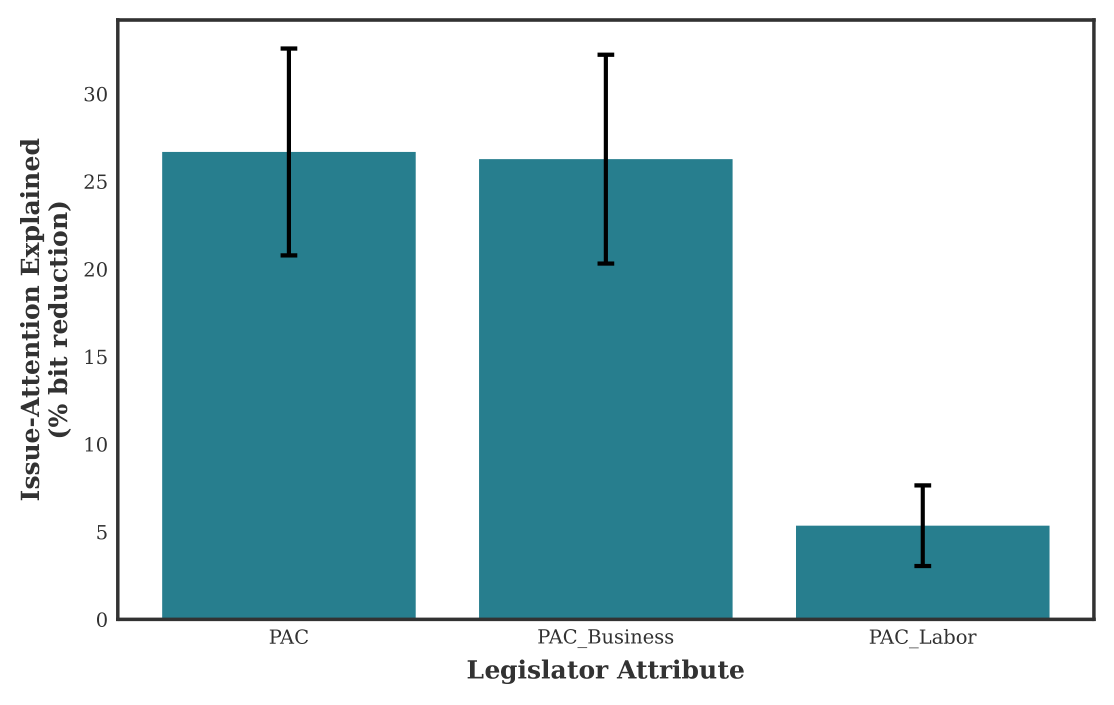

Supplement: S22 Fig — (TIF) [file pone.0291169.s037.tif]

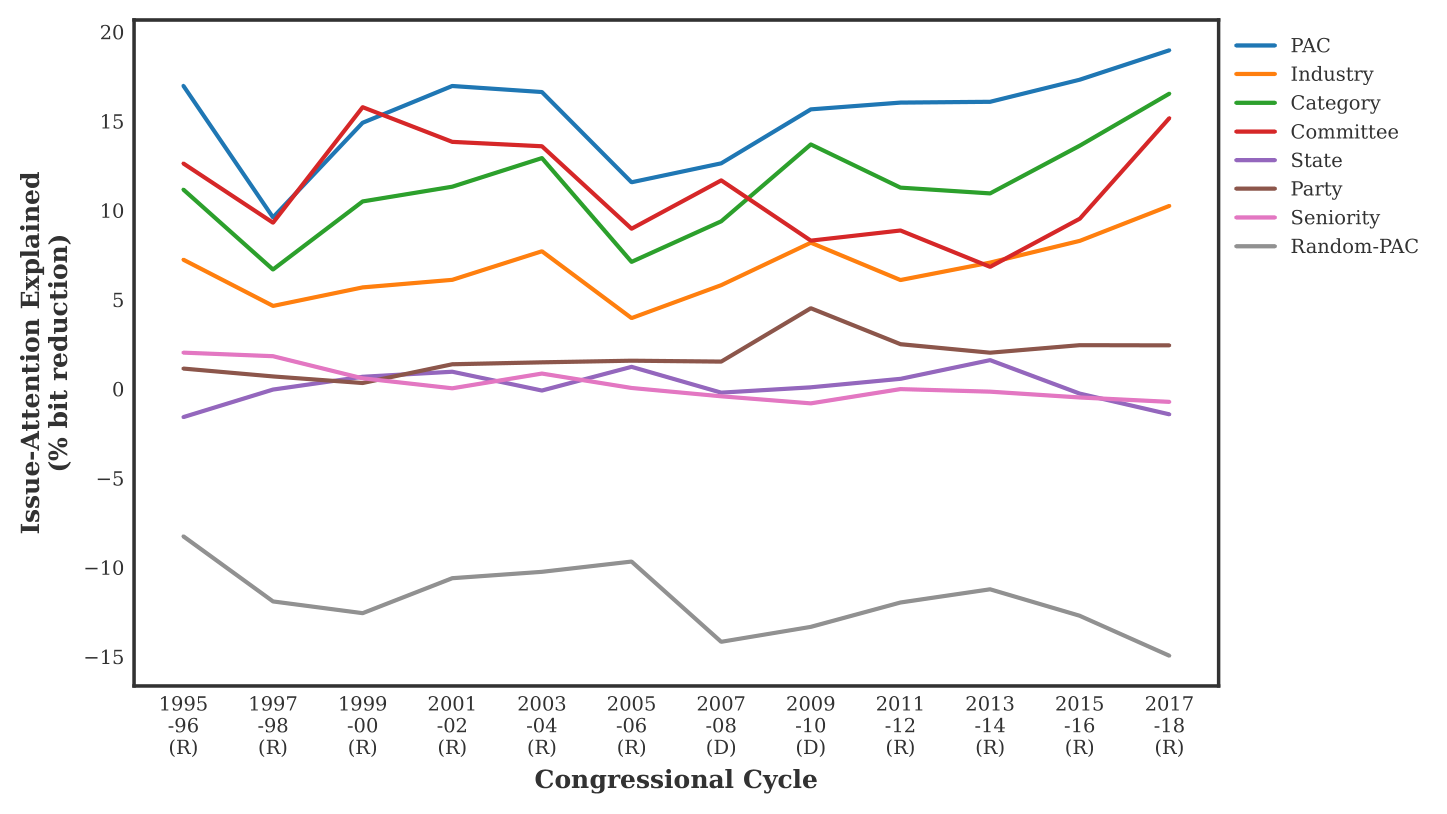

Supplement: S23 Fig — (TIF) [file pone.0291169.s038.tif]

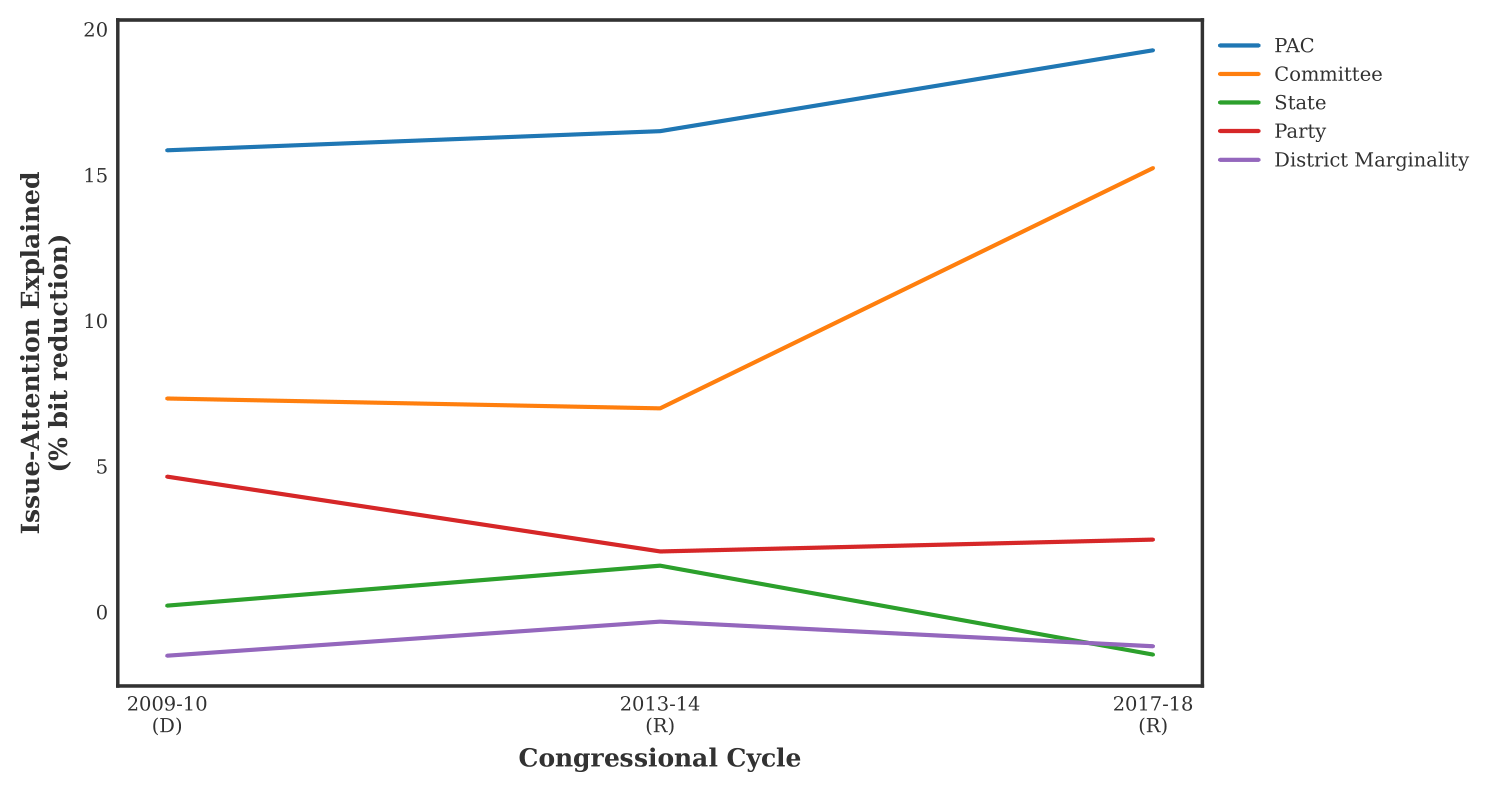

Supplement: S24 Fig — The comparison is done across three different Congresses—2009–10, 2013–14, and 2017–18—and uses data on district-level voting for US presidential candidates immediately preceding the particular Congress under consideration. (TIF) [file pone.0291169.s039.tif]
