# Supplementary material for: Donor activity is associated with US legislators’ attention to political issues
Source: PLoS One. 2023 Sep 20;18(9):e0291169. doi: 10.1371/journal.pone.0291169 (PMC10511130; doi:10.1371/journal.pone.0291169)
Supplement: S1 Table — The attributes are used to predict the 758 X 60 legislator-level average topic proportions in our multinomial regularized logistic regression approach. (PDF) [file pone.0291169.s040.pdf]

S1 Table. Dimensions or sizes of the various legislator attributes. The attributes are used to predict the 758 X 60 legislator-level average topic proportions in our multinomial regularized logistic regression approach.

**Legislator Attribute**	**Dimensionality**
PAC	$758X1002$
Industry	$758X72$
Category	$758X250$
Committee	$758X94$
State	$758X56$
Party	$758X3$
Random-PAC	$758X1002$
