# Supplementary material for: Donor activity is associated with US legislators’ attention to political issues
Source: PLoS One. 2023 Sep 20;18(9):e0291169. doi: 10.1371/journal.pone.0291169 (PMC10511130; doi:10.1371/journal.pone.0291169)
Supplement: S2 Table — The first value in each dimensionality is the number of speakers selected in that cycle, and the second value is the number of features (for the legislator attribute or model indicated in the column title). Note that the y for each of the regression models (averaged topic distribution for speakers) is going to be (number of speakers, 30). (PDF) [file pone.0291169.s041.pdf]

S2 Table. The dimensionality of each of our legislator attributes for the modeling done separately for each congressional cycle. The first value in each dimensionality is the number of speakers selected in that cycle, and the second value is the number of features (for the legislator attribute or model indicated in the column title). Note that the y for each of the regression models (averaged topic distribution for speakers) is going to be (number of speakers, 30).

	**PAC**	**Industry**	**Category**	**Committee**	**State**	**Party**	**Random-PAC**
**1995-96**	(312, 617)	(312, 69)	(312, 215)	(312, 81)	(312, 50)	(312, 3)	(312, 617)
**1997-98**	(382, 648)	(382, 69)	(382, 218)	(382, 85)	(382, 52)	(382, 3)	(382, 648)
**1999-00**	(415, 694)	(415, 70)	(415, 225)	(415, 87)	(415, 54)	(415, 3)	(415, 694)
**2001-02**	(416, 743)	(416, 71)	(416, 228)	(416, 85)	(416, 54)	(416, 3)	(416, 743)
**2003-04**	(418, 754)	(418, 72)	(418, 230)	(418, 87)	(418, 54)	(418, 3)	(418, 754)
**2005-06**	(421, 800)	(421, 72)	(421, 235)	(421, 91)	(421, 54)	(421, 3)	(421, 800)
**2007-08**	(416, 823)	(416, 72)	(416, 241)	(416, 92)	(416, 53)	(416, 2)	(416, 823)
**2009-10**	(396, 835)	(396, 71)	(396, 243)	(396, 93)	(396, 55)	(396, 2)	(396, 835)
**2011-12**	(401, 875)	(401, 71)	(401, 247)	(401, 92)	(401, 54)	(401, 2)	(401, 875)
**2013-14**	(402, 854)	(402, 71)	(402, 247)	(402, 89)	(402, 53)	(402, 2)	(402, 854)
**2015-16**	(355, 837)	(355, 71)	(355, 247)	(355, 89)	(355, 52)	(355, 2)	(355, 837)
**2017-18**	(303, 810)	(303, 71)	(303, 246)	(303, 89)	(303, 50)	(303, 2)	(303, 810)
