# Supplementary material for: Donor activity is associated with US legislators’ attention to political issues
Source: PLoS One. 2023 Sep 20;18(9):e0291169. doi: 10.1371/journal.pone.0291169 (PMC10511130; doi:10.1371/journal.pone.0291169)
Supplement: S3 Table — Note that experts created labels for topics using not just the top terms, but also the top documents (floor speeches) for the topic given by the topic model. And contextualizing info such as sector, industry, and the industrial category was provided when asking for association ratings on the 1–3 Likert scale. (PDF) [file pone.0291169.s042.pdf]

S3 Table. Examples of expert-provided issue labels for topics. Note that experts created labels for topics using not just the top terms, but also the top documents (floor speeches) for the topic given by the topic model. And contextualizing info such as sector, industry, and the industrial category was provided when asking for association ratings on the 1-3 Likert scale.

**Issue (top terms by topic model)**	israel, terrorism, terrorist, attacks, attack, terrorists, east, peace, middle, september, middle_east, the_middle_east, threat, terror, syria	education, college, students, programs, training, student, workforce, institutions, opportunities, loans, skills, university, colleges, grants, universities	medical, patients, doctors, hospital, patient, hospitals, doctor, quality, providers, physicians, physician, medicine, centers, emergency, managed
**Label (Expert 1)**	Foreign Policy (Middle East)	Education (Higher ed)	Healthcare
**Label (Expert 2)**	Terrorism	Education	Healthcare
**(Top PACs) #1**	US Beet Sugar Assn	Sears Holdings Corp	Society for Vascular Surgery
**#2**	Viacom International	Bridgepoint Education	American Psychiatric Assn
**#3**	Greenberg Traurig LLP	National Education Assn	Cooperative of American Physicians
**#4**	AECOM US	McDonald's Corp	Society of Thoracic Surgeons
**#5**	Fluor Corp	TIAA	American Pharmacists Assn
**#6**	Pizza Hut Franchisees Assn	Prudential Financial	American Assn/Oral & Maxillofacial Surg
**#7**	American Express	NelNet Inc	American Assn of Neurological Surgeons
**#8**	American International Group	Navient Corp	American Academy of Family Physicians
**#9**	Exelis Inc	American Speech-Language-Hearing Assn	American Society of Plastic Surgeons
**#10**	American Society of Interventional Pain Physicians	Career Education Colleges & Universities	American Academy of Otolaryngology
