# Supplementary material for: Donor activity is associated with US legislators’ attention to political issues
Source: PLoS One. 2023 Sep 20;18(9):e0291169. doi: 10.1371/journal.pone.0291169 (PMC10511130; doi:10.1371/journal.pone.0291169)
Supplement: S4 Table — The best settings found for PAC were also used for Random-PAC. (PDF) [file pone.0291169.s043.pdf]

S4 Table. Values for optimal hyperparameter settings for the multinomial logistic regression model. The best settings found for *PAC* were also used for *Random-PAC*.

	**PAC**	**Industry**	**Category**	**Committee**	**State**	**Party**
α	0.00001	0.000005	0.000001	0.00005	0.00005	0.00005
λ	0.01	0.005	0.005	0.001	0.001	0.0
η	1	1	1	1	1	1
