# Supplementary material for: Donor activity is associated with US legislators’ attention to political issues
Source: PLoS One. 2023 Sep 20;18(9):e0291169. doi: 10.1371/journal.pone.0291169 (PMC10511130; doi:10.1371/journal.pone.0291169)
Supplement: S5 Table — p < 0.05 (boldfaced) indicates significantly better results for the PAC attribute against the other attribute for that congressional session. (PDF) [file pone.0291169.s044.pdf]

S5 Table. Statistical significance test results using Mann-Whitney U test, comparing 30-fold cross-validation results for $\boldsymbol{PAC}$ against other legislator attributes. $p < 0.05$ (boldfaced) indicates significantly better results for the PAC attribute against the other attribute for that congressional session.

Congress	PAC >Committee	PAC >Category	PAC >Industry	PAC >State	PAC >Party
1995-96	**0.0399**	**0.0040**	**0.0000**	**0.0**	**0.0**
1997-98	0.4036	**0.0362**	**0.0007**	**0.0**	**0.0**
1999-00	0.4853	**0.0018**	**0.0000**	**0.0**	**0.0**
2001-02	**0.0412**	**0.0013**	**0.0000**	**0.0**	**0.0**
2003-04	0.0560	**0.0099**	**0.0000**	**0.0**	**0.0**
2005-06	**0.0386**	**0.0026**	**0.0000**	**0.0**	**0.0**
2007-08	0.2998	**0.0146**	**0.0001**	**0.0**	**0.0**
2009-10	**0.0001**	0.0747	**0.0000**	**0.0**	**0.0**
2011-12	**0.0001**	**0.0013**	**0.0000**	**0.0**	**0.0**
2013-14	**0.0000**	**0.0015**	**0.0000**	**0.0**	**0.0**
2015-16	**0.0000**	**0.0116**	**0.0000**	**0.0**	**0.0**
2017-18	0.0812	0.1259	**0.0002**	**0.0**	**0.0**
