# Supplementary material for: Donor activity is associated with US legislators’ attention to political issues
Source: PLoS One. 2023 Sep 20;18(9):e0291169. doi: 10.1371/journal.pone.0291169 (PMC10511130; doi:10.1371/journal.pone.0291169)
Supplement: S6 Table — p < 0.05 (boldfaced) indicates significantly better results for the Committee attribute against the other attribute for that congressional session. (PDF) [file pone.0291169.s045.pdf]

S6 Table. Statistical significance test results using Mann-Whitney U test, comparing 30-fold cross-validation results for *Committee* against other legislator attributes. $p < 0.05$ (boldfaced) indicates significantly better results for the *Committee* attribute against the other attribute for that congressional session.

Congress	Committee >PAC	Committee >Category	Committee >Industry	Committee >State	Committee >Party
1995-96	0.9614	0.2459	**0.0169**	**0.0000**	**0.0000**
1997-98	0.6021	0.0869	**0.0038**	**0.0000**	**0.0000**
1999-00	0.5206	**0.0043**	**0.0000**	**0.0000**	**0.0000**
2001-02	0.9601	0.1452	**0.0000**	**0.0000**	**0.0000**
2003-04	0.9457	0.4094	**0.0045**	**0.0000**	**0.0000**
2005-06	0.9626	0.0881	**0.0001**	**0.0000**	**0.0000**
2007-08	0.7053	0.1141	**0.0021**	**0.0000**	**0.0000**
2009-10	0.9999	0.9988	0.4617	**0.0000**	**0.0049**
2011-12	0.9999	0.9096	0.0706	**0.0000**	**0.0002**
2013-14	1.0000	0.9964	0.5733	**0.0001**	**0.0001**
2015-16	1.0000	0.9834	0.2188	**0.0000**	**0.0000**
2017-18	0.9210	0.6633	**0.0130**	**0.0000**	**0.0000**
