# Supplementary material for: Donor activity is associated with US legislators’ attention to political issues
Source: PLoS One. 2023 Sep 20;18(9):e0291169. doi: 10.1371/journal.pone.0291169 (PMC10511130; doi:10.1371/journal.pone.0291169)
Supplement: S7 Table — After selecting relevant issue-PAC pairs with potential temporal connection for the cycle: some examples of speeches made on the topic and cases of the relevant PAC donating an amount much higher than their mean donation amount during that congressional cycle to the legislator giving that speech within a particular time window around the speech. (PDF) [file pone.0291169.s046.pdf]

S7 Table. Examples of meaningful (non-random) relevant speech and donation events occurring in close proximity. After selecting relevant issue-PAC pairs with potential temporal connection for the cycle: some examples of speeches made on the topic and cases of the relevant PAC donating an amount much higher than their mean donation amount during that congressional cycle to the legislator giving that speech within a particular time window around the speech.

PAC name	Topic label (expert 1/ expert 2)	Speech text snippet	Legislator (Affiliation), Speech Date	Donation to legislator (Amount, Date)	Mean (std. dev.) donation amount for the donor in the election cycle
General Dynamics	Defense/ Military	Mr. Speaker, I thank the gentleman for yielding me time. Mr. Speaker, I just wanted to state, so my colleagues will understand very clearly, and I think the gentleman from Pennsylvania [Mr. Weldon] made the point, that this President does not want to defend the United States against incoming ballistic missiles. That was his major objection to this bill, along with the idea that he also wants to have the right to delegate to foreign commanders the command of U.S. troops. We are now going to enter a period in which it is important for Members of this House who feel that defense is important to enter a full-court press this year to develop defenses against incoming ballistic missiles, both for the people of the United States and for our troops in theater. We are going to do this. The President has given up his most solemn responsibility, and that is to defend the people of the United States of America, and he is denied that responsibility in this bill.	Duncan L. Hunter (R-CA) on 1996-01-24	2000 USD, 1996-01-21	720 USD (±470 USD)
AFLAC Inc (Accident and health supplemental insurance company;)	Economic/ Economy (jobs)	Mr. Speaker, as a result of our arcane and complicated pension laws, 70 million workers have no pension plan. Unfortunately, Americans who work in small businesses are much less likely to have pension coverage than those who work for larger companies. Among companies with fewer than 100 employees, as many as 80% of the workforce have no retirement savings plan available to them. The primary cause: small business owners find the cost and complexity of setting up and maintaining retirement plans to be overwhelming. So last year, Congress passed the Portman-Cardin pension reforms to help workers save for their future and enable small businesses to offer pension plans to their employees. The changes we made streamline and simplify the complex rules governing our pension system to ensure meaningful coverage of small business employees. They will reduce the administrative burden on small businesses and provide incentives to help them establish plans for their workers, including cutting the IRS user fee small businesses have to pay to establish a pension plan and lowering premiums small businesses pay for their defined benefit plans to make that option more attractive …	Nancy Johnson (R - CT) on 2002-06-21	5000 USD, 2002-06-14	1690 USD (±1220 USD)
BAE Systems	International Security/ Foreign Policy	Mr. Chairman, I thank the gentleman from Wisconsin for yielding time to me. First let me say that I agree very much, this is an American, this is a NATO conflict. We in this House should speak with one voice and not be putting it on political terms. I feel very, very deeply about this. I support this bill. At the end of the day, I support this bill. It is a major step toward my goal of making this the year of the troops, the year in which we recognize the needs of those who serve in uniform. I also support it because it ensures that our military has more than adequate resources to carry out the Kosovo air campaign. It bolsters the military readiness of our forces in the Balkan theater and the Armed Forces as a whole. It provides the resources to help address the tragic humanitarian situation in Kosovo. The basis of this bill was a $6 billion administration request in emergency funding. The request was based on four categories, military operations in and around Kosovo, Kosovar refugee relief, munitions and readiness munitions, and Desert Thunder and Desert Fox military operations. In addition to the administration's original request, our colleagues on the Committee on Appropriations have seen fit to add to the President's request, both to the humanitarian request and the matter request. There are some problems that our colleagues had on the Committee on Appropriations, and they have tried to address them. They have added certain categories. Mr. Chairman, allow me to comment on two major additions to the original request. First, this bill sends the right signal to our men and women in uniform by providing $1.8 billion to fund the administration's military pay and retirement package, of course, conditioned upon the enactment of authorizing legislation through our Committee on Armed Services. Second, this bill provides for $1.1 billion in unrequested funds for overseas military construction in Europe and Southeast Asia. The inclusion of these projects is similar to the inclusion of the administration's pay and retirement package.	Ike Skelton (D - MO) on 1999-05-06	3000 USD, 1999-05-07	890 USD (±620 USD)
