# Supplementary material for: Donor activity is associated with US legislators’ attention to political issues
Source: PLoS One. 2023 Sep 20;18(9):e0291169. doi: 10.1371/journal.pone.0291169 (PMC10511130; doi:10.1371/journal.pone.0291169)
Supplement: S8 Table — For a PAC (BAE Systems) and an issue (International Security/Foreign Policy) that our model (as well as experts) deem related to one another, we find that in a particular congressional cycle (1995–96), there is a significant temporal connection between this PAC and the issue, i.e., this PAC donates a significant amount closer to speeches on the particular issue of international security and foreign policy in 1995–96 than for speeches that are not on this issue. In particular, the legislator—Norm Dicks (D-WA)—emerged as a frequent recipient of such temporally significant donations by this PAC, and a simple search on a search engine reveals that this connection is backed by what is known to journalists and validated by real-world knowledge in a straightforward manner (the last three columns of the table). (PDF) [file pone.0291169.s047.pdf]

S8 Table. An example of the kind of real-world relationships that the outputs of our modeling and analysis can capture automatically. For a PAC (BAE Systems) and an issue (International Security/Foreign Policy) that our model (as well as experts) deem related to one another, we find that in a particular congressional cycle (1995-96), there is a significant temporal connection between this PAC and the issue, *i.e.*, this PAC donates a significant amount closer to speeches on the particular issue of international security and foreign policy in 1995-96 than for speeches that are not on this issue. In particular, the legislator — Norm Dicks (D-WA) — emerged as a frequent recipient of such temporally significant donations by this PAC, and a simple search on a search engine reveals that this connection is backed by what is known to journalists and validated by real-world knowledge in a straightforward manner (the last three columns of the table).

**PAC name**	**Topic label (Expert 1/ Expert 2)**	**Legislator (Affiliation)**	**Article URL (for face validity)**	**Article headline (for face validity)**	**Relevant text snippet from the article (for face validity)**
BAE Systems	International Security/ Foreign Policy	Norm Dicks (D-WA)	https://armscontrolcenter.org/ norm-dicks-the-next-likely-chairman-of-the-house-defense-appropriations subcommittee/	Norm Dicks, the next likely Chairman of the House Defense Appropriations Subcommittee	"The differences [from Democrats] usually occur on defense spending and foreign policy issues, where he is often more hawkish than the party norm."
