# Supplementary material for: Donor activity is associated with US legislators’ attention to political issues
Source: PLoS One. 2023 Sep 20;18(9):e0291169. doi: 10.1371/journal.pone.0291169 (PMC10511130; doi:10.1371/journal.pone.0291169)
Supplement: S9 Table — Or a PAC (National Assn of Federally Insured Credit Unions) and an issue (Finance/Financial regulation) that our model (as well as experts) deem related to one another, we find that in two different congressional cycles (2009–10 and 2015–16), there is a significant temporal connection between this PAC and the issue, i.e., this PAC donates a significant amount closer to speeches on the particular issue of finance or financial regulation in both 2009–10 and 2015–16 than for speeches that are not on this issue. In particular, the legislator—Ed Royce (R-CA)—emerged as the recipient of such temporally significant donations by this PAC in both cycles, and a simple search on a search engine reveals that this connection is backed by what is known to journalists and validated by real-world knowledge in a straightforward manner (the last three columns of the table). (PDF) [file pone.0291169.s048.pdf]

S9 Table. An example of the kind of real-world relationships that the outputs of our modeling and analysis can capture automatically. or a PAC (National Assn of Federally Insured Credit Unions) and an issue (Finance/Financial regulation) that our model (as well as experts) deem related to one another, we find that in two different congressional cycles (2009-10 and 2015-16), there is a significant temporal connection between this PAC and the issue, *i.e.*, this PAC donates a significant amount closer to speeches on the particular issue of finance or financial regulation in both 2009-10 and 2015-16 than for speeches that are not on this issue. In particular, the legislator — Ed Royce (R-CA) — emerged as the recipient of such temporally significant donations by this PAC in both cycles, and a simple search on a search engine reveals that this connection is backed by what is known to journalists and validated by real-world knowledge in a straightforward manner (the last three columns of the table).

**PAC name**	**Topic label (Expert 1/ Expert 2)**	**Legislator (Affiliation)**	**Article URL (for face validity)**	**Article headline (for face validity)**	**Relevant text snippet from the article (for face validity)**
National Assn of Federally Insured Credit Unions	Finance/ Financial Regulation	Ed Royce (R-CA)	https://www.americanbanker.com/creditunions/news/big-questions-for-credit-unions-as-royce-retires-from-house/	Big questions for CUs as Royce retires from House	"... one exit may sting the credit union movement more than the rest. U.S. Rep. Ed Royce (R-CA) ... who has earned a reputation for being a credit union champion, having introduced a number of pro-credit union bills during his tenure."
